# Unsupervised Segmentation of 3D Microvascular Photoacoustic Images Using Deep Generative Learning

**DOI:** 10.1002/advs.202402195

**Published:** 2024-06-23

**Authors:** Paul W. Sweeney, Lina Hacker, Thierry L. Lefebvre, Emma L. Brown, Janek Gröhl, Sarah E. Bohndiek

**Affiliations:** ^1^ Cancer Research UK Cambridge Institute University of Cambridge Robinson Way Cambridge CB2 0RE UK; ^2^ Department of Physics University of Cambridge JJ Thomson Avenue Cambridge CB3 0HE UK

**Keywords:** blood vessels, deep learning, generative, photoacoustics, segmentation, unsupervised

## Abstract

Mesoscopic photoacoustic imaging (PAI) enables label‐free visualization of vascular networks in tissues with high contrast and resolution. Segmenting these networks from 3D PAI data and interpreting their physiological and pathological significance is crucial yet challenging due to the time‐consuming and error‐prone nature of current methods. Deep learning offers a potential solution; however, supervised analysis frameworks typically require human‐annotated ground‐truth labels. To address this, an unsupervised image‐to‐image translation deep learning model is introduced, the Vessel Segmentation Generative Adversarial Network (VAN‐GAN). VAN‐GAN integrates synthetic blood vessel networks that closely resemble real‐life anatomy into its training process and learns to replicate the underlying physics of the PAI system in order to learn how to segment vasculature from 3D photoacoustic images. Applied to a diverse range of in silico, in vitro, and in vivo data, including patient‐derived breast cancer xenograft models and 3D clinical angiograms, VAN‐GAN demonstrates its capability to facilitate accurate and unbiased segmentation of 3D vascular networks. By leveraging synthetic data, VAN‐GAN reduces the reliance on manual labeling, thus lowering the barrier to entry for high‐quality blood vessel segmentation (F1 score: VAN‐GAN vs. U‐Net = 0.84 vs. 0.87) and enhancing preclinical and clinical research into vascular structure and function.

## Graphical abstract

This study introduces VAN‐GAN, an unsupervised deep learning model for 3D vascular network segmentation in mesoscopic photoacoustic imaging. By integrating synthetic blood vessel networks and advanced training techniques, VAN‐GAN demonstrates accurate and unbiased segmentation across in silico, in vitro, and in vivo datasets, including clinical angiograms, reducing reliance on manual labeling and minimizing bias in vascular research.

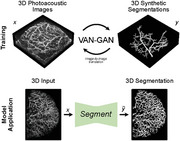

## 1 Introduction

Mesoscopic photoacoustic imaging (PAI) provides high‐resolution, contrast‐rich images of vascular structures in vivo based on the absorption of light by haemoglobin. These images reveal subtle changes in vascular architecture in tissues, which have demonstrated importance in diagnosis and staging of a range of diseases, from diabetes to oncology.^[^ ^1^, ^2^ ^]^ To quantify disease status, segmenting microvascular networks from 3D image volumes is vital, however, the development of segmentation frameworks has not kept pace with the rapid advancements in imaging technology. While deep learning offers a promising solution,^[^ ^3^ ^]^ its effective application in this field is fraught with complexities.

Current supervised segmentation frameworks^[^ ^4^, ^5^, ^6^ ^]^ typically require matched pairs of image volumes and human‐annotated ground‐truth feature masks. Manually generating vascular labels is time‐consuming so is typically limited to a small number of image pairs. Furthermore, human annotation is error‐prone, particularly in complex pathological tissues like tumours^[^ ^7^, ^8^ ^]^ or in the presence of imaging artefacts, where interpretation of what constitutes a blood vessel can vary between experts.

Supervised blood vessel segmentation models have been demonstrated based on annotated datasets in 2D retinal fluorescein angiograms^[^ ^9^, ^10^ ^]^ or 3D multiphoton images of brain vasculature.^[^ ^11^, ^12^, ^13^ ^]^ Efforts have also been made to develop semi‐supervised methods, which reduce the paired dataset size required^[^ ^14^, ^15^, ^16^, ^17^, ^18^ ^]^ or minimize ground‐truth ambiguity.^[^ ^18^ ^]^ Given the challenges associated with manual labeling, unsupervised learning has naturally gained special attention for image segmentation tasks.^[^ ^19^ ^]^ In particular, cycle‐consistent generative adversarial networks (CycleGAN) can be used to transform images from a imaging (source) domain to a segmentation (target) domain. By assuming an underlying relationship between each domain, CycleGAN performs style transfer between datasets of unpaired images by allowing two neural network translators to be trained in a constrained unsupervised and competitive manner. Modified formulations of CycleGAN have accurately segmented biomedical images,^[^ ^20^, ^21^, ^22^, ^23^, ^24^ ^]^ including cell nuclei from 3D fluorescence microscopy^[^ ^25^ ^]^ and blood vessels in 2D X‐ray angiograms.^[^ ^26^ ^]^

To date, the application of deep learning methods in PAI has primarily focused on image reconstruction^[^ ^27^, ^28^, ^29^ ^]^ or bridging the domain gap between simulations and experiments.^[^ ^30^, ^31^ ^]^ Supervised methods have not only explored the task of blood vessel segmentation from 2D photoacoustic images^[^ ^32^, ^33^, ^34^, ^35^, ^36^ ^]^ but have also shown promise in addressing the complexities involved in segmenting 3D images, as recent work has demonstrated the potential of combining synthetic data and manual annotations for effective supervised segmentation of 3D photoacoustic images.^[^ ^37^ ^]^ Nonetheless, the transition to unsupervised methods in 3D PAI has been challenging due to artefacts arising from low signal‐to‐noise ratio (SNR)^[^ ^38^ ^]^ or specific excitation and detection geometries,^[^ ^39^ ^]^ which severely limit their effectiveness. Whilst innovative unsupervised approaches have been applied in other 3D imaging modalities,^[^ ^40^, ^41^ ^]^ there remains a need for tailored unsupervised models that overcome specific challenges in 3D PAI.

In this study, we introduce VAN‐GAN ( *V* *essel Segment* *a* *tio* *n* *G* *enerative* *A* *dversarial* *N* *etwork*), an innovative unsupervised deep learning model tailored to 3D vascular network segmentation in mesoscopic PAI. VAN‐GAN eliminates the need for human‐annotated labels, employing image‐to‐image translation to adeptly transform PAI volumes into precise 3D vascular segmentation maps. Our model leverages a synthetic dataset enriched with a variety of vascular morphologies, further enhanced by advanced 3D deep residual U‐Net generators and cycle‐consistency loss functions. A significant breakthrough of VAN‐GAN is its ability to segment vascular networks from tissue types beyond those included in its training dataset, showcasing adaptability and precision even in the low signal‐to‐noise ratio environments typical of PAI. The challenges of manual labeling, the ambiguity in ground‐truth data, and the susceptibility to imaging artefacts have critically limited the applicability of traditional segmentation models in preclinical and clinical PAI settings. Demonstrating successful application to a diverse range of in silico and in vivo PAI datasets, VAN‐GAN not only outperforms conventional supervised methods but also exhibits remarkable robustness against user bias and common PAI artefacts. VAN‐GAN's capabilities significantly extend the boundaries of what has been traditionally achievable in the field, offering a robust and scalable solution that could enhance PAI deployed for both preclinical research and clinical diagnostics.

## 2 Results

### 2.1 Advancing 3D Vessel Segmentation in Photoacoustic Imaging Using VAN‐GAN

**Figure 1:**
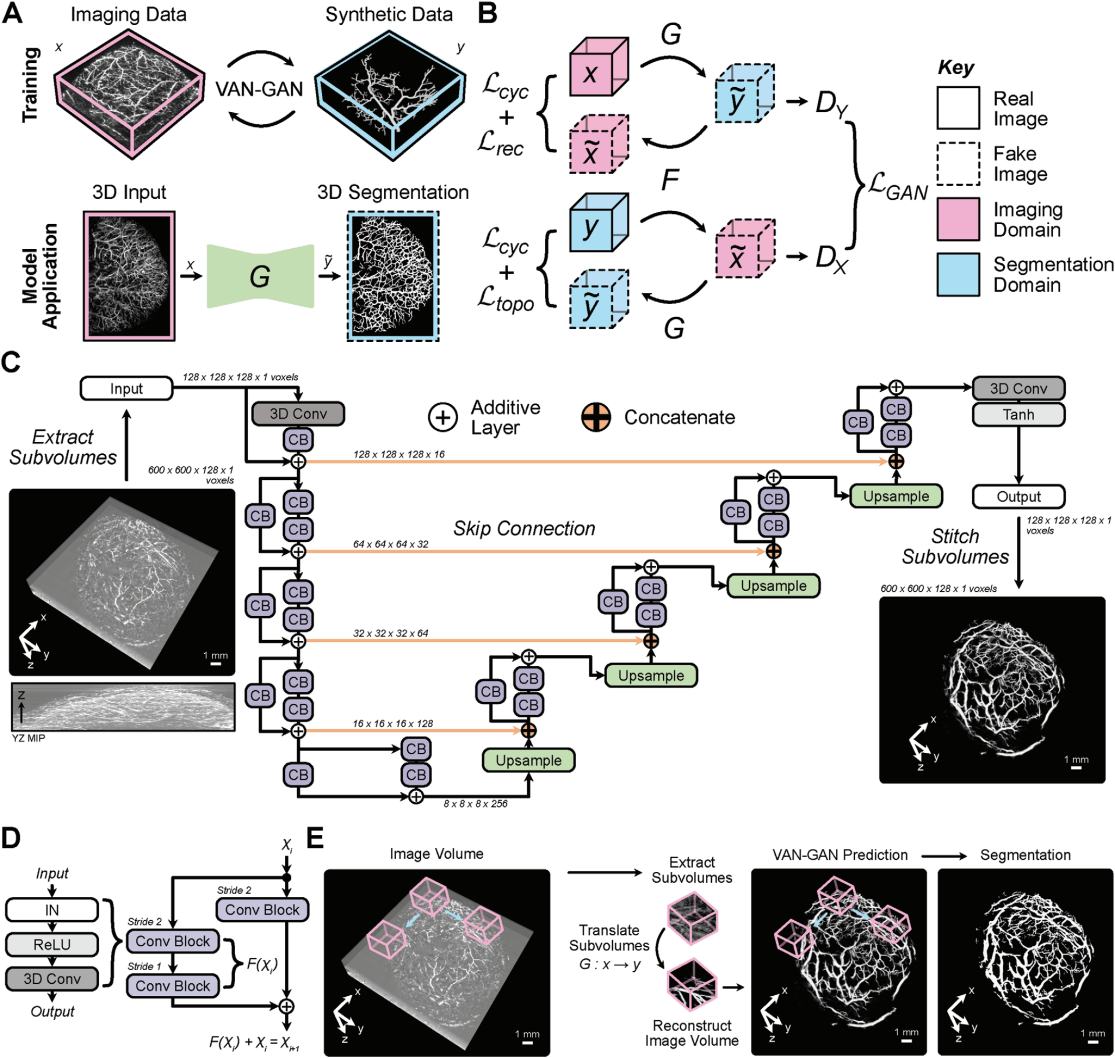
Vessel segmentation generative adversarial network (VAN‐GAN) architecture for unsupervised segmentation of blood vessels in photoacoustic imaging (PAI) volumes. A) The training and application process of VAN‐GAN utilizes two unpaired datasets (real PAI volumes, *x*, and synthetic blood vessels, *y*) to train the segmentor, *G*, for real‐world use. B) VAN‐GAN adapts the cycleGAN model and learns mappings between imaging (*x*) and segmentation (*y*) domains ($G:x\to \tilde{y}$, $F:y\to \tilde{x}$) using additional reconstruction, $L_{rec}$, and topological, $L_{topo}$, cycle‐consistency constraints. C) A 3D deep residual U‐Net generator architectures are employed. An example, PAI subvolume input (left) and segmentation output (right) is shown. D) Standard residual units are employed for each generator. E) A sliding window approach is used to form a probability map that is binarised to create the final segmentation mask. CB = convolutional block; MIP = maximal intensity projection; IN = instance normalization.

VAN‐GAN is engineered to learn mappings between the imaging domain and segmentation domain, thereby training a segmenter that is adept for real‐world PAI applications (**Figure** **1**a). The imaging domain comprises PAI volumes, either generated through physics‐driven simulations or acquired experimentally. Uniquely, the segmentation domain utilizes computer‐generated 3D branching blood vessels, stochastically created using mathematical formalisms, to mimic branching vascular architectures (see Experimental Section). Throughout our study, the dataset for the segmentation domain remains consistent, and during the training of VAN‐GAN, the datasets for imaging and segmentation domains are treated as unpaired.

VAN‐GAN builds upon the CycleGAN model^[^ ^42^ ^]^ with several key enhancements to address the specific challenges of PAI (see Experimental Section and Supporting Information for full details): 3D deep residual U‐Net generators, optimized loss functions with regularisation, and a sliding window approach for large 3D images. The generator architecture integrates a 3D U‐Net^[^ ^4^, ^5^ ^]^ with deep residual learning^[^ ^43^ ^]^ (Figure 1c,d, Table S1, Supporting Information) whereas the discriminator uses a 3D PatchGAN discriminator architecture (see Table S2, Supporting Information).^[^ ^44^ ^]^

The additional cycle‐consistency loss functions to optimize performance. These include a structural similarity reconstruction loss, which is applied to both real and reconstructed images in the imaging domain, and a spatial topological constraint, applied to the real and reconstructed images in the segmentation domain to preserve tubular structures^[^ ^45^ ^]^ (Figure 1b). Notably, each generator optimizes its own respective total cycle consistency loss. Consequently, VAN‐GAN gains the flexibility to delineate intricate details in the images and explore innovative segmentation approaches without facing immediate penalties when translating back to the imaging domain. To further improve training stability and regularization, random Gaussian noise is strategically introduced to the discriminator inputs and layers,^[^ ^46^, ^47^ ^]^, which helps in preventing over‐fitting and enhances generalisability.

Finally, VAN‐GAN adopts a sliding window approach during training, strategically addressing the computational demands inherent in processing full 3D volumes by enabling it to efficiently handle large datasets by segmenting them into manageable sub‐volumes. As the window strides over the image volume, any looping vascular network structures emulate branching patterns. The sliding window method equips VAN‐GAN to generalize across complex vascular architectures not explicitly present in the training dataset.

### 2.2 VAN‐GAN Rivals Supervised Learning in Segmenting Photoacoustic Images

**Figure 2:**
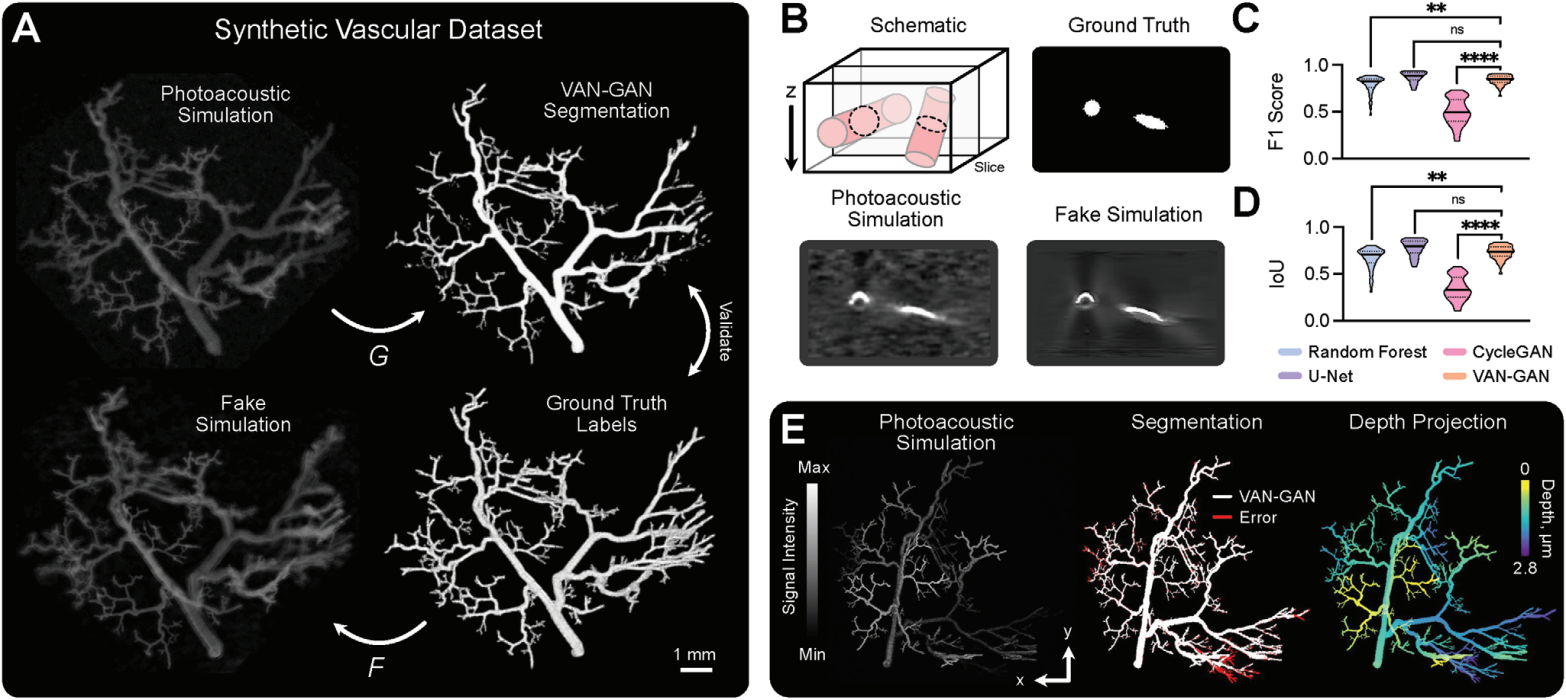
VAN‐GAN performance on synthetic photoacoustic data. A) Illustration of the paired dataset, consisting of synthetic vessel structures that form ground‐truth labels and corresponding physics‐driven PAI simulations. B) Fake simulation images generated by generator *F* reproduce artefacts (present in our simulations) that are inherent to mesoscopic PAI and influence vessel shape and continuity. The example illustrates an artefact arising from limited angular coverage of the illumination fibres, which means the circular vessel cross‐section appears as an arc in PAI. Segmentation metric distributions compare performance of a random forest pixel classifier, U‐Net, CycleGAN and VAN‐GAN: C) F1 Score; and D) Intersection over Union (IoU). E) An example of (left) a photoacoustic simulation, (middle) the corresponding VAN‐GAN segmentation labels and error with respect to the ground‐truth, and (right) a color‐coded depth projection of the network. Data in (C,D) are represented by truncated violin plots with interquartile range (dotted) and median (solid) shown. Statistical significance indicated by * (*P* < 0.05), ** (*P* < 0.01), *** (*P* < 0.001), and **** (*P* < 0.0001). *ns* = no significance.

We first tested VAN‐GAN using physics‐driven simulations to virtually emulate PAI, giving the stochastic 3D branching vessel structures from VAN‐GAN's segmentation domain as simulation inputs (see Experimental Section, and Figures S1 and S2, Supporting Information). In essence, this created a paired in silico dataset of PAI volumes and their segmentations, which enables direct validation of VAN‐GAN's segmentation performance (**Figure** **2**a).

During training, VAN‐GAN perceived images from both the imaging and segmentation domains as unpaired and thus processed them in an unsupervised manner. Then during testing, the paired dataset enabled us to perform an ablation study to quantitatively evaluate the impact of key VAN‐GAN components on the segmentation accuracy via their systematic removal from the model. The ablation study indicated that VAN‐GAN was able to generate fake PAI volumes that included typical photoacoustic artefacts, such as those arising from limited illumination and detection views, as well as shadowing from overlying absorbers^[^ ^39^ ^]^ (Figure 2b). We found that omission of discriminator noise and reconstruction loss in the reduced models impeded learning of these image features, whereas the addition of the topological loss provides a greater constraint to improve segmentation of vascular structures (**Table** **1**). The full VAN‐GAN model did not achieve the highest sensitivity score (0.834), which is attributed to model adaptation to noise annealing and balancing multiple loss functions. This may lead to under‐prioritizing the detection of subtle features crucial for high sensitivity. However, the full model achieved the highest F1 Score (0.842) and IoU (0.730), offering a more comprehensive view of performance by reducing false positives and favoring specificity in vessel detection for improved segmentation.

**Table 1:** VAN‐GAN ablation study. The following components were systematically removed to evaluate the performance of our model in their absence: 1) random Gaussian noise added to discriminator inputs and convolution layers (*Noise*); 2) a perceptual constraint on cycle reconstructed photoacoustic images (*Rec*.); and 3) a spatial and topological constrained on cycle reconstructed segmentation images (*Topo*.). In the *Base* setup, all these components are ablated. The inclusion of all three components, *Base + Noise + Rec. Loss + Top. Loss*, gives the full VAN‐GAN model. Note, specificity excluded due to ≈0.999 values for all methods.

Method	F1 Score	IoU	Sensitivity
Base	0.793	0.661	0.768
Base + Noise	0.434	0.282	0.303
Base + Rec. Loss	0.675	0.514	0.694
Base + Top. Loss	0.772	0.633	**0.864**
Base + Noise + Rec. Loss	0.581	0.411	0.634
Base + Noise + Top. Loss	0.760	0.618	0.793
Base + Rec. Loss + Top. Loss	0.675	0.518	0.770
Base + Noise + Rec. Loss + Top. Loss	**0.842**	**0.730**	0.834

VAN‐GAN was then benchmarked using the paired synthetic data against other deep‐learning models, selected for their relevance, and proven effectiveness in image segmentation within the scope of PAI. A comparison with CycleGAN^[^ ^42^ ^]^ was important, given its foundational role in, and architectural similarities to VAN‐GAN, allowing us to highlight the benefits of specific enhancements and optimizations introduced in VAN‐GAN. For supervised methods, a random forest (RF) pixel classifier and 3D U‐Net were chosen. The RF method was embedded within the open‐source package ilastik,^[^ ^48^ ^]^ which is particularly advantageous because it can be trained using incomplete labels. Additionally, we included a 3D U‐Net,^[^ ^5^ ^]^ regarded as the gold standard for medical image segmentation as the architecture can capture and utilise contextual information at multiple resolutions. CycleGAN and VAN‐GAN treated the dataset as unpaired during training, whereas full feature labels were supplied to the RF and U‐Net (see Supporting Information). The dataset consisted of 449 paired images with 10% assigned for testing and the remaining 90% split between training (80%) and validation (20%).

The results show good performance by VAN‐GAN with, for example, mean F1 Score / IoU of 0.842 / 0.778, compared to the RF (0.792 / 0.665), U‐Net (0.873 / 0.778) and CycleGAN (0.502 / 0.346) (**Table** **2**). Significant differences between metrics were found when comparing VAN‐GAN to RF and CycleGAN (Figure 2c,d) and due to its poor performance, CycleGAN was excluded from all further analyzes. Comparison to U‐Net found there was no significant difference in performance in these fully labeled synthetic datasets, however, in an in vivo context, ground‐truth annotations are partial at best and we found the U‐Net trained on synthetic data lacked generalisability to in vivo data (Figure S3, Supporting Information), hence the U‐Net model was also excluded from all further analyses. RF methods are typically able to maintain performance with only partially labeled data, so RF was used as a comparator in subsequent analyses in the in vivo setting.

**Table 2:** Comparison of segmentation performance using PAI simulations. F1 Score, intersection over union (IoU), sensitivity, and specificity were calculated on the testing dataset. RF = random forest pixel classifier and the highest metric score per ground‐truth is shown in bold. Statistical significance between VAN‐GAN is indicated by * (*P* < 0.05), ** (*P* < 0.01), *** (*P* < 0.001), and **** (*P* < 0.0001). If blank, no significance was found compared to VAN‐GAN.

Method	F1 Score	IoU	Sensitivity	Specificity
VAN‐GAN	0.842	0.730	0.834	0.999
CycleGAN	0.502^****^	0.346^****^	0.558^****^	0.997^****^
RF	0.792^**^	0.665^**^	0.748^**^	0.999
U‐Net	**0.873**	**0.778**	**0.877**	**0.999**

Qualitative examination of VAN‐GAN segmentation predictions showed errors were largely confined to the smallest vessels, which exhibited relatively low SNR as they were furthest from the simulated light‐source in the tissue (Figure 2e).

### 2.3 VAN‐GAN Avoids User Bias Arising from Human Annotations

**Figure 3:**
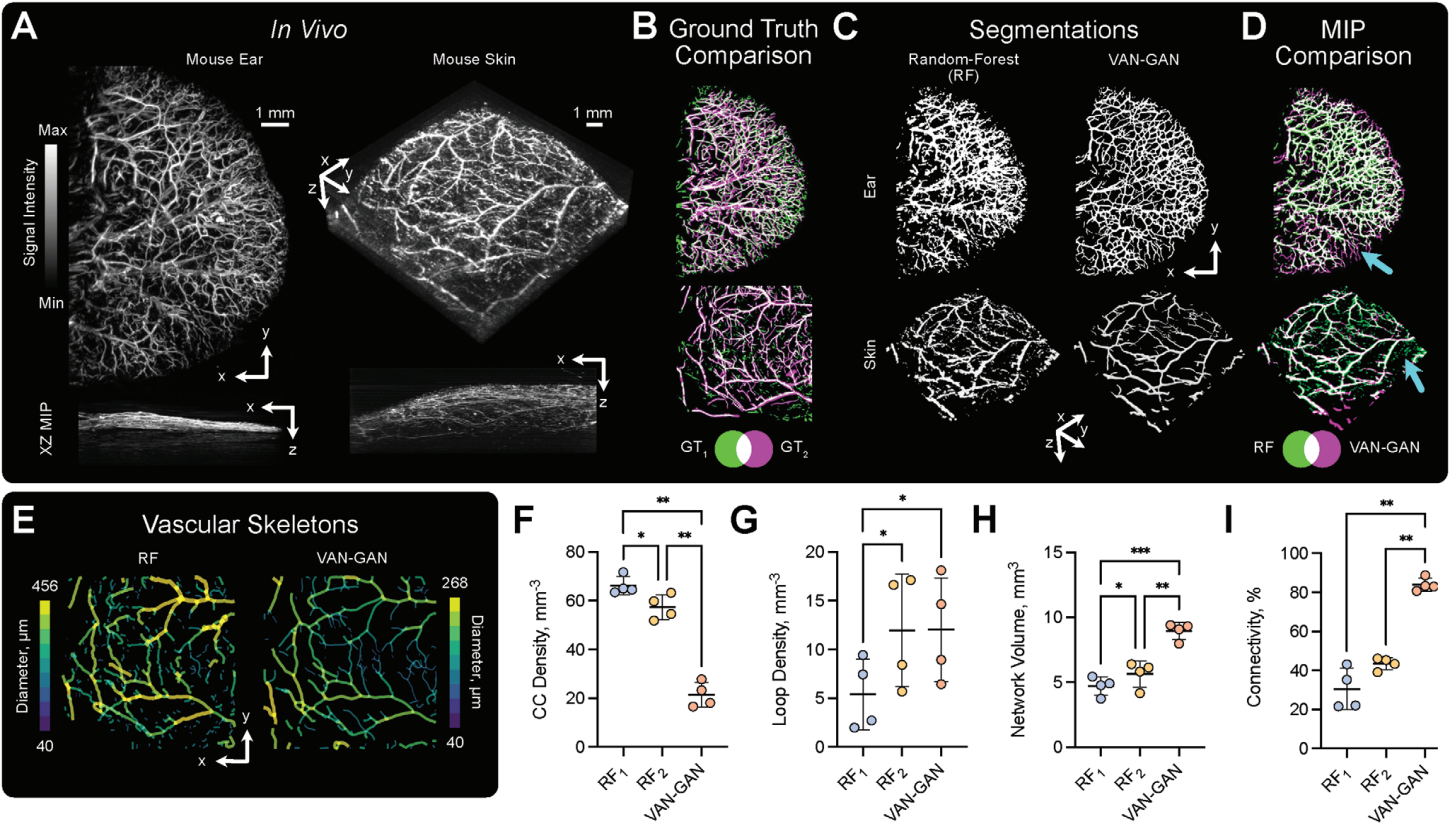
Unsupervised vascular segmentation overcomes user variation in human annotations. A) Mouse ear and skin imaged in vivo using PAI. B) Ground‐truths for XY maximal intensity projections (MIPs) were generated by two experts (indicated by subscripts *1* and *2*). Visualizations indicate variability between each expert for the ear (top) and skin (bottom). C) 3D segmentation masks predicted using RF (left) and VAN‐GAN (right) for datasets shown in (A,B). D) Comparison of MIPs generated from the 3D segmentation labels shown in (C) with arrows indicating segmented noise and vessel discontinuities using RF. E) 2D projections of 3D vascular skeletons created from the two segmentation methods applied to mouse skin. Variability between individual users RF classifiers and VAN‐GAN on the skeletonized mouse skin is indicated for a range of statistical and vascular descriptors: F) connected component (CC) density (the number of subnetworks normalized against tissue volume, 1mm^−3^); G) loop density (the number of vessel network loops normalised against tissue volume, 1/mm^3^); (H) network volume (mm^3^); and (I) connectivity (the volume of the largest subnetwork normalised against total network volume, %);. Mean and standard deviation of data are shown in (F–I). Statistical significance indicated by * (*P* < 0.05), ** (*P* < 0.01), *** (*P* < 0.001), and **** (*P* < 0.0001). MIP = maximal intensity projection.

Given the need for human annotations in supervised training, we sought to compare the impact of expert variability on this process. First, two experts independently labeled blood vessels on 2D maximum intensity projections (MIPs) generated from 3D in vivo PAI data from mouse ears (*n* = 1) and skin (*n* = 4) (**Figure** **3**a). MIPs were specifically employed to mitigate prevalent surface illumination artefacts that typically lead to only the upper part of absorbing structures being excited and detected, which has been shown to result in inaccurate 3D segmentations by human annotators.^[^ ^38^ ^]^

Comparing the two expert annotations, substantial differences were observed between their segmentations both qualitatively (Figure 3b) and quantitatively (F1 Score: 0.667/0.569, IoU: 0.501/0.397, Specificity: 0.916/0.892 and Sensitivity: 0.754/0.659 for ear / skin), highlighting the challenges in ground‐truth label creation in real imaging data. These expert annotators then independently trained separate 3D RF models for vessel segmentation in mouse ear and skin datasets. Comparing these models showed no bias toward respective expert labels; each RF model performed poorly against the 2D annotations (for example, F1 Score: 0.668 / 0.613 for ear / skin for the RF model and ground‐truths created by expert 1 ‐ Table S5, Supporting Information). VAN‐GAN's accuracy was also low overall (F1 Score: 0.649 / 0.529 for ear / skin compared against ground‐truths labeled by expert 1), however, it was more robust to segmentation of background noise as closer scrutiny revealed limitations in RF, such as artefact segmentation and discontinuities in segmented vessels (Figure 3c,d).

To analyze more comprehensively the 3D morphology of vascular networks segmented by the RF and VAN‐GAN models, we skeletonized the 3D vessel masks (Figure 3e) and calculated a set of vascular descriptors based on topological data analysis (see Experimental Section). Focusing on the mouse skin, we found that the number of vascular subnetworks (or connected components) and network loops between RF_1_ and RF_2_ were significantly different (Figure 3f,g). These analyzes highlight a critical flaw in using supervised segmentation models ‐ their performance is inherently limited by the quality of training data. Independent training by different expert users can lead to systematically varied segmentation outcomes (Figure 3H), which could bias study interpretation. By comparison, VAN‐GAN predicts more larger interconnected vascular networks, with the largest subnetwork on average forming 84.0% of the total subnetwork volume (Figure 3h,i). These results indicate that VAN‐GAN segments more well‐connected networks compared to RF, which aligns better with the expectations of the biological network in healthy skin.

### 2.4 VAN‐GAN is More Robust to Artefacts Compared to Human Annotations

**Figure 4:**
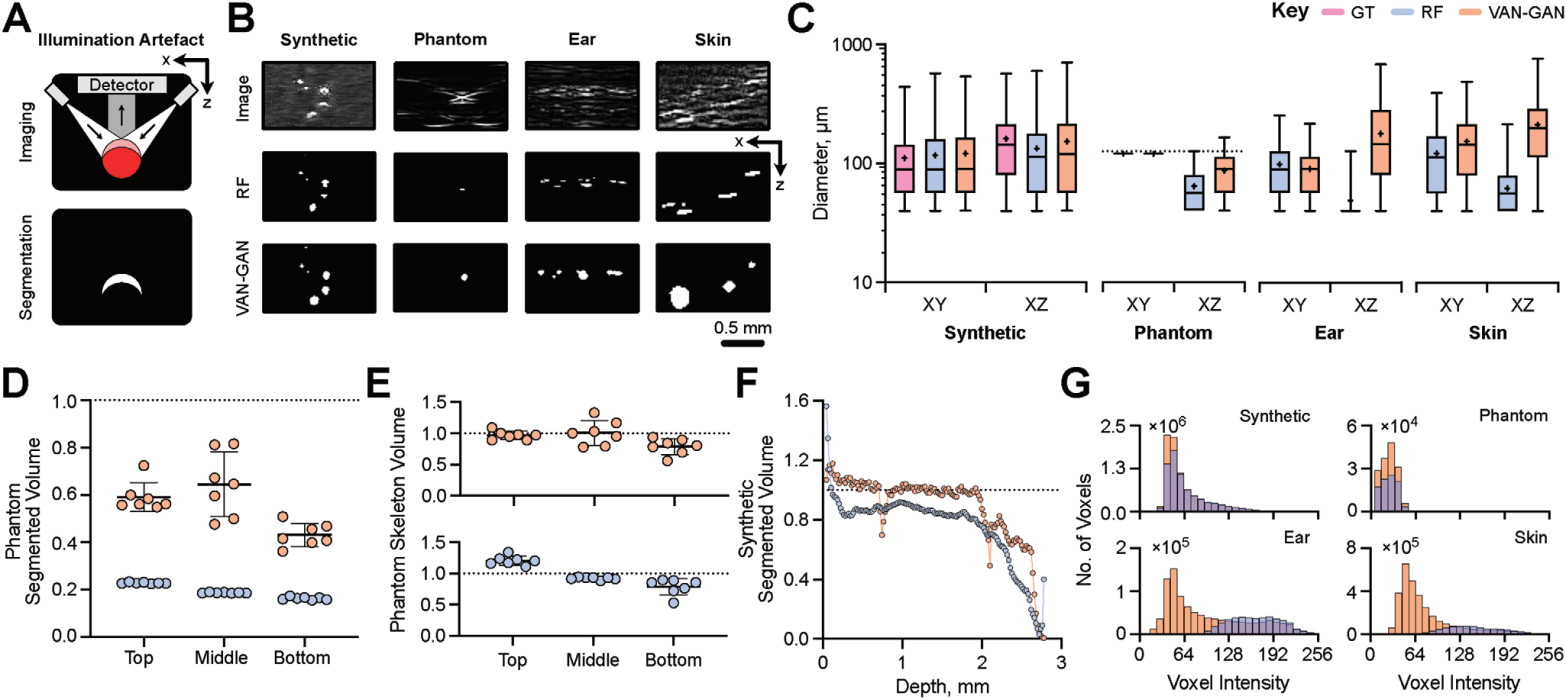
VAN‐GAN overcomes image artefacts in photoacoustic mesoscopy. A) The illumination artefact in photoacoustic mesoscopy results in the incorrect segmentation of the top surface of blood vessels due to lower signals from underlying regions. B) Example image subsections highlighting the illumination artefact in each imaging domain dataset and corresponding random forest pixel classifier (RF) and VAN‐GAN segmentations. RF models for phantom, ear and skin segmentation were trained by an expert whereas the RF model for the synthetic data was trained using known computer‐generated ground‐truths. C) Diameters in the XY and XZ directions calculated for each dataset. For the string phantom, the known string volume is indicated by the dotted line. Computed string phantom volumes from D) 3D segmented images and E) skeletonised strings normalized against the ground‐truth value. F) Normalized segmented volume for our synthetic dataset with respect to tissue depth. G) Histograms of signal intensities for each voxel segmented by each method and for each dataset: synthetic vasculatures, in situ string phantoms and in vivo mouse ear and skin datasets. Data in (C) is given by box and whisker plots that display the 25^*th*^, 50^*th*^, and 75^*th*^ quartiles, and the interquartile range. Mean and standard deviation of data are shown in (D) and (E).

Several imaging artefacts impede segmentation performance in PAI. Illumination artefacts, resulting from limited light coverage, cause objects to appear flattened in images (**Figure** **4**a), leading human users to annotate only the brighter top surfaces of blood vessels. In our paired synthetic dataset, we addressed this by training the supervised RF with full ground‐truth labels, enabling it to learn the complete range of signal intensities expected from the reference structures. Training an RF segmenter with these paired synthetic data resulted in more accurate, axisymmetric vessel segmentation than that achieved by an RF model specialized for experimental in vivo datasets and trained by a human annotator (Figure 4b,c).

In contrast, VAN‐GAN effectively accounts for the entire signal intensity range across vessel walls and lumens in both in silico and in vivo datasets. When comparing diameters in the XZ‐plane, VAN‐GAN's predictions were closer to ground‐truth, outperforming the RF model (Figure 4b,c). Accuracy was further validated using photoacoustic string phantoms, which consist of three non‐overlapping strings of known size located at three separate depths parallel to the imaged surface.^[^ ^39^ ^]^ Although VAN‐GAN was not specifically trained on string phantom images, it more accurately predicted string diameters in the XZ‐plane than the RF model, demonstrating its robustness and versatility to illumination artefacts.

Further artefacts arise in PAI due to depth‐dependent SNR. VAN‐GAN demonstrated a consistent ability to accurately predict string volumes at various depths (Figure 4d). Both VAN‐GAN and RF models showed improved network volume predictions after applying skeletonisation, which presumes axisymmetric vessels (Figure 4e). On the synthetic dataset, VAN‐GAN consistently outperformed RF, especially when depth exceeded 2 mm (Figure 4f). A detailed analysis of segmented voxel signal intensities revealed a tendency for VAN‐GAN to segment more low‐intensity voxels across all datasets, a trend that became more pronounced for in vivo data (Figure 4g). Compared to RF models with their limited receptive fields, VAN‐GAN appears superior in handling complex spatially‐varying background noise and can better learn intricate feature representations.

### 2.5 VAN‐GAN Segments Vascular Topologies Beyond the Training Dataset

PAI is vital for monitoring blood vessel evolution in tumours, which present unique segmentation challenges due to their heterogeneous nature.^[^ ^49^, ^50^ ^]^ Unlike physiological tissues, tumour vascular architecture is chaotic with varying diameters, lengths, and inter‐connectivity across various spatial scales. These features are absent in VAN‐GAN's synthetic segmentation domain dataset since the method used to generate branching structures inherently leads to regular and predictable patterns.

**Figure 5:**
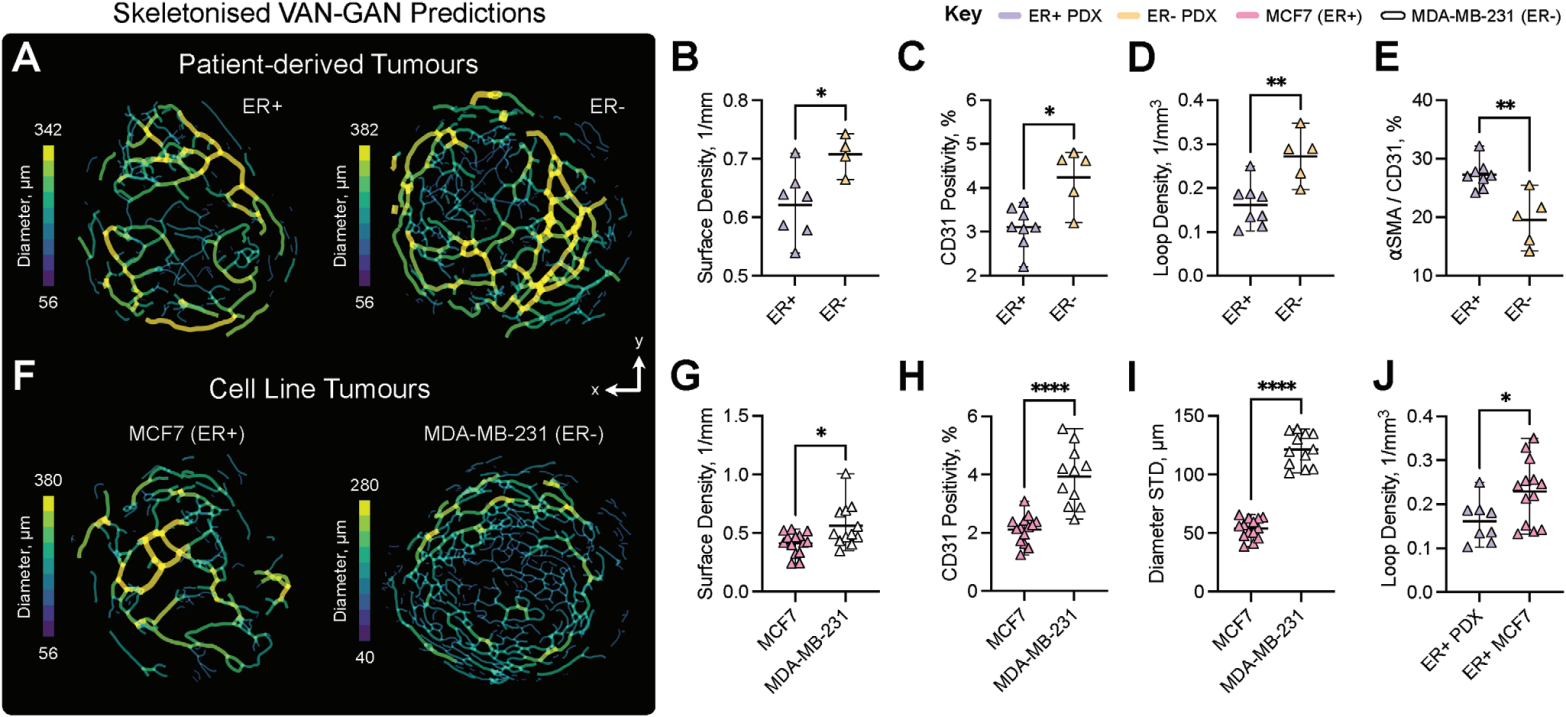
VAN‐GAN enables quantification of pathological vascular architecture. A) Vascular skeletons for oestrogen receptor negative (ER‐, left) and positive (ER+, right) breast cancer patient‐derived xenograft tumours. A comparison of metrics for ER‐ (top) and ER+ (bottom): B) vessel surface density (1/mm), C) CD31 positivity (staining area with respect to tumour area, %), (D) vessel loop density (1mm^−3^) and E) α‐smooth muscle actin (αSMA) colocalization with CD31 staining (%). F) Vascular skeletons of MCF7 (left) and MDA‐MB‐231 (right) breast cancer tumours. VAN‐GAN metrics for MCF7 and MDA‐MB‐231 tumours: G) vessel surface density (1/mm), H) CD31 positivity and I) standard deviation (STD) of vessel diameters (µm). J) A comparison of vessel loop density (1mm^−3^) in ER+ models. Mean and standard deviation of data are shown in (C–F) and (H–K). Statistical significance indicated by * (*P* < 0.05), ** (*P* < 0.01), *** (*P* < 0.001), and **** (*P* < 0.0001).

To evaluate the ability of VAN‐GAN to segment complex pathological vascular networks, datasets of 3D images of oestrogen receptor positive (ER+) and negative (ER‐) breast cancer tumours derived from both patient‐derived xenograft models^[^ ^38^, ^51^ ^]^ and cell lines (MCF7 and MDA‐MB‐231, respectively) were used. Segmentations made by VAN‐GAN allowed hypothesised structural differences in vasculature between the ER+ and ER‐ subtypes to be identified in both tumour types (**Figure** **5**a and Supporting Information). In the PDX tumours, the ER‐ tumours exhibited significantly higher vessel surface area density with respect to tumour volume (*P* < 0.05, Figure 5b). The ex vivo immunohistochemistry (IHC) analysis of CD31 staining, an endothelial cell marker, cross‐validated this finding, as the ER‐ tumours showed significantly greater CD31 positivity (*P* < 0.05, Figure 5c). Additionally, VAN‐GAN indicated ER‐ tumours displayed a higher density of vascular looping structures (*P* < 0.01, Figure 5d) and reduced vessel lengths (*P* < 0.01), which could indicate a more immature vascular network compared to ER+. IHC staining of α‐smooth muscle actin (αSMA), a pericyte and smooth muscle marker, colocalized with CD31, supported this finding as ER‐ tumours showed significantly lower positivity (*P* < 0.01, Figure 5e).

VAN‐GAN also showed distinct features between the cell‐line derived MCF7 (ER+) and MDA‐MB‐231 (ER‐) tumours (Figure 5f) that were cross‐validated by IHC. In our analysis, we quantified the blood vessel network surface area relative to the tumour volume using VAN‐GAN segmentations. These measurements revealed similar trends (*P* < 0.05, Figure 5g), with ER‐ MDA‐MB‐231 tumors exhibiting significantly elevated levels of CD31 expression (*P* < 0.0001, Figure 5h). αSMA staining also indicated lower positivity for ER‐ MDA‐MB‐231 tumours (*P* < 0.01) but no significance in vessel loops or lengths between groups was found between each oestrogen receptor group. Vessels in the ER‐ MDA‐MB‐231 tumours exhibited greater heterogeneity (vessel diameters standard deviation, *P* < 0.0001, Figure 5i), in contrast to the pattern observed in PDX tumours where greater heterogeneity was noted in ER+ tumours (*P* < 0.05). Observed differences between PDX and cell‐line derived tumours were underscored by a significant difference in looping structure between ER+ models (*P* < 0.05, Figure 5j), highlighting divergence in vascular maturity. The ER+ PDX tumours were likely at a more advanced stage of vascular development compared to ER+ MCF7 tumours.

The close relationship of the topological descriptors extracted from VAN‐GAN segmentations to IHC analyses of the same tumours, together with common trends across different tumour models, indicate that VAN‐GAN is able to accurate segment complex vasculatures that exceed the constraints imposed by the synthetic segmentation data used in its training.

### 2.6 VAN‐GAN Demonstrates Cross‐Platform Adaptability in Human Clinical Imaging

**Figure 6:**
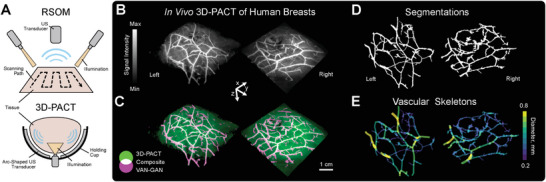
VAN‐GAN showcases cross‐platform compatibility with clinical photoacoustic imaging. A) A schematic of raster‐scanning photoacoustic imaging (RSOM), the mesoscopic system used, compared to the clinical 3D photoacoustic computed tomography (3D‐PACT) systems.^[^ ^56^ ^]^ (B) In vivo 3D‐PACT images of human breasts. C) Superimposition of 3D‐PACT images with VAN‐GAN segmentations. D) Segmentations and E) skeletons of the largest vascular networks identified by VAN‐GAN.

PAI has demonstrated promise in early‐stage clinical trials for various pathologies,^[^ ^52^, ^53^ ^]^ particularly in oncology, where the FDA has approved the use of PAI in the breast cancer diagnostic pathway.^[^ ^54^ ^]^ PAI excels in delineating the angiographic anatomy of breast tumours, providing critical insights into their vascular structures.^[^ ^55^ ^]^ To demonstrate the potential clinical value of VAN‐GAN, we undertook a proof‐of‐concept study on PAI angiograms of two healthy human breasts acquired in vivo using 3D photoacoustic computed tomography (3D‐PACT).^[^ ^52^ ^]^ Compared to mesoscopic PAI, the tomographic data, acquired independently by another laboratory, utilized a lower spatial resolution of 50 × 50 × 50µm^3^ and a larger field‐of‐view. 3D‐PACT can also introduce further modality‐specific imaging artefacts due to device design (**Figure** **6**a). Given limited data availability, the VAN‐GAN model pretrained on in vivo mesoscopic preclinical images was applied to the clinical data without additional training.

The vascular segmentations predicted by VAN‐GAN closely matched the 3D‐PACT angiograms (Figure 6b,c), which allowed for the extraction of the largest segmented vascular network from each breast (Figure 6d), facilitating the quantification of vascular structures (Figure 6d). Some 3D‐PACT features were absent in the training dataset, ranging from movement artefacts to signal variations caused by differences in transducer geometry, which likely contributed to the observed errors in segmentation. Expanding the training dataset with tomographic images could reduce these inaccuracies, enhancing VAN‐GAN's ability to quantify anatomical features in clinical PAI.

## 3 Discussion

Here, we introduced VAN‐GAN, an innovative deep learning model that segments 3D vascular networks imaged using PAI mesoscopy. For mesoscopic PAI, human annotations are time consuming and laborious due to depth‐dependent SNR and subtle imaging artefacts, which make it particularly challenging to label pathological tissues such as tumours. Independent ground‐truth labeling by two expert users in our study showed substantial discrepancies in both their segmentations and the vascular topology parameters measured from subsequent skeletonisations, highlighting the potential for detrimental impact of user bias on quantification of blood vessel networks.

VAN‐GAN adeptly navigates these difficulties in multiple ways. First, VAN‐GAN is a novel approach that builds on the foundation of CycleGAN by integrating 3D deep residual U‐Net generators and bespoke cycle‐consistency loss functions and discriminator noise, fully leveraging the power of unsupervised learning for PAI image segmentation. These additional elements were found through an ablation study to enhance the capability of VAN‐GAN in segmenting intricate vascular structures from synthetic PAI volumes, leading to VAN‐GAN surpassing traditional supervised methods and rivalling the gold standard U‐Net.

Second, VAN‐GAN is able to handle complex imaging artefacts arising from the geometry of the PAI system, demonstrating robustness in both synthetic and real‐world datasets. VAN‐GAN errors were generally confined to only the smallest vessels. Importantly, VAN‐GAN provided realistic quantification of vessel lumens, which otherwise appear flattened in supervised segmentations due to illumination artefacts; VAN‐GAN restored the segmenation that would be expected based on reference structures (in phantoms) and maintained lumen patency through to healthy ear and skin tissues. VAN‐GAN also demonstrated greater robustness to depth‐dependent SNR, segmenting at a greater depth than supervised methods and providing the most accurate quantifications with depth of the tested methods.

VAN‐GAN provides biologically relevant segmentations for in vivo preclinical PAI data, showing more interconnected and larger vascular networks in the healthy ear and skin than other methods. VAN‐GAN also extended directly to application in pathological tissues, such as patient‐derived breast cancer, even though the complex chaotic architectures associated with these tissue types were absent from the synthetic dataset used for training. Topological data analysis of skeletonized vascular networks derived from VAN‐GAN segmentations showed biological findings consistent with IHC analysis conducted on ex vivo sections. Taken together, these three main findings demonstrate strengths of VAN‐GAN to significantly boost the precision and reliability of vascular segmentation in PAI, while emphasising the versatility of the approach.

VAN‐GAN has demonstrated impressive capabilities, however, it is important to consider the limitations of its training. Reliance on synthetic data, though effective, may in future need further adaptation to encompass the full diversity of real‐world vascular structures, particularly when considering application to human imaging data. Such adaptations could include developing more complex simulations to create more realistic vasculatures for simulation or integrating manual 3D labels from a diverse range of imaging techniques into the training data. Additionally, extending applicability to a wider range of tissue types and chromophores, beyond those included in its initial training, would be important. All of the animal studies undertaken here were in nude mice, which lack skin pigmentation, however, skin tone is a consideration that is gaining greater attention in the PAI community^[^ ^57^ ^]^ and data from a range of skin tones would be needed to maximize applicability of VAN‐GAN in future.

Finally, VAN‐GAN has demonstrated potential as a complementary tool in clinical PAI diagnostics. Its capability to adapt to clinical 3D photoacoustic computed tomography (3D‐PACT) without additional training is particularly promising, illustrating the model's versatility and potential for integration with existing clinical workflows. As the availability of clinical photoacoustic images for model refinement increases, VAN‐GAN's cross‐platform functionality underscores its readiness for clinical deployment.

Moving forward, the potential applications of VAN‐GAN extend beyond PAI. A key focus area for enhancement is the optimization of training schemes and loss function weightings, which are crucial for ensuring the generalisability and efficacy of the model across diverse imaging contexts. Integrating explainable AI components into VAN‐GAN is also essential to increase transparency and build trust in clinical settings, enhancing its potential for clinical deployment. Merging VAN‐GAN with open‐source bioimage platforms would also be important to democratise access to advanced segmentation tools, fostering wider adoption and application in the life sciences. Integration such as this not only aligns with the trend towards accessible, high‐quality image analysis but also opens new avenues for research and clinical applications by providing consistent and unbiased results.

In conclusion, VAN‐GAN sets a new precedent in the segmentation of 3D microvascular networks in mesoscopic PAI. By reducing the reliance on manual labeling and leveraging synthetic data, our approach promises to lower the barrier to entry for high‐quality blood vessel segmentation, leading to more robust and consistent characterization of vascular structures. VAN‐GAN could thus not only deepen our understanding of tumour vascular architectures but also pave the way for the discovery of novel vascular‐targeted therapeutics and improvement of diagnostic accuracy across clinical applications.

## 4 Experimental Section

The following details architecture and training methodology of VAN‐GAN, in addition to describing image synthesis and preprocessing. VAN‐GAN was implemented using Tensorflow^[^ ^58^ ^]^ with Keras backend^[^ ^59^ ^]^ and Tensorflow Addons, along with Tensorflow‐MRI.^[^ ^60^ ^]^ The model was trained on either: 1) a Dell Precision 7920T with a Dual Intel Xeon Gold 5120 CPU with 128GB RAM and two NVIDIA Quadro GV100 32GB GPUs with NVLink; or 2) a custom built workstation with a Intel Xeon Gold 5220 CPU with 128GB RAM and four NVIDIA RTX A6000 48GB GPUs paired with NVLinks. The optimal model for each dataset was selected for application based on qualitative image evaluation of generated images from the test set and a comprehensive analysis of minima for each loss function (see Figures S4–S6, Supporting Information).

### VAN‐GAN Architecture

VAN‐GAN generators used a modified version of a deep residual U‐Net architecture^[^ ^61^ ^]^ (see Table S2, Supporting Information), which integrates residual units into a 3D U‐Net architecture to ease training and facilitate information propagation through the network without degradation. For the latter, it was important to use low‐level details and retain high‐resolution semantic information to improve segmentation performance.^[^ ^4^, ^5^, ^61^, ^62^ ^]^ The convolutional block sequence included instance normalization,^[^ ^63^ ^]^ ReLU activation,^[^ ^64^ ^]^ and 3D convolutions, with reflection padding to minimise boundary artefacts.^[^ ^42^, ^65^ ^]^ Both the generator input and output tensor shapes were 128 × 128 × 128 × 1 (*depth* × *width* × *height* × *channel*).

The generators consisted of three parts: encoder, bridge, and decoder^[^ ^61^ ^]^ (Figure 1c). The encoder and decoder feature four layers with residual units (Figure 1d) connected by skip connections. The convolutional block sequence includes instance normalisation,^[^ ^63^ ^]^ ReLU activation,^[^ ^64^ ^]^ and 3D convolution, with reflection padding to minimize boundary artefacts.^[^ ^42^, ^65^ ^]^

The discriminators used a five layer PatchGAN architecture^[^ ^44^ ^]^ (see Table S3, Supporting Information). Each layer was composed of a 3D convolutional layer, instance normalization,^[^ ^63^ ^]^ leaky ReLU,^[^ ^66^ ^]^ and spatial dropout^[^ ^67^ ^]^ (a rate of 20% and excluded from first and final layers). Similarly to the generators, reflection padding was also used prior to convolution layers to reduce feature map artefacts.^[^ ^65^ ^]^ Further, for additional regularization and to limit unstable behavior of VAN‐GAN during training, random Gaussian noise was added to real or fake inputs to the discriminator^[^ ^46^, ^47^ ^]^ and for every proceeding layer prior to convolution blocks^[^ ^47^ ^]^ (Table S4, Supporting Information).

### VAN‐GAN Optimisation

The goal of VAN‐GAN is to learn the mapping functions *G*: *X* → *Y* and *F*: *Y* → *X* between the imaging, *X*, and segmentation, *Y*, domains. In the VAN‐GAN model, each generator was designed to minimize its own respective cycle‐consistency loss, rather than collectively minimising a total cycle‐consistency loss as in CycleGAN.^[^ ^42^ ^]^ Consequently, for each domain transformation the corresponding generator was responsible for reducing the discrepancy between the original input and cycle reconstructed output.

*L*_1_‐norm was utilized for forward cycle‐consistency, i.e., *x* → *G*(*x*) → *F*(*G*(*x*)) ≈ *x*:

(1) $$L_{cyc}^{G}\left(G,F,x\right)=E_{x∼p_{data}\left(x\right)}\left[||F\left(G\left(x\right)\right)-{x||}_{1}\right]$$

and binary cross‐entropy for backward cycle‐consistency, i.e., *y* → *F*(*y*) → *G*(*F*(*y*)) ≈ *y*:

(2) $$L_{cyc}^{F}\left(G,F,y\right)=-E_{y∼p_{data}\left(y\right)}\left\{y\log\left[G(F(y))\right]+\left(1-y\right)\log\left[1-G(F(y))\right]\right\}$$

Separating total cycle consistency components enables more specialized optimization of the generators by reducing the potential for conflicting domain transformation objectives, particularly given the imaging and segmentation domains are highly disparate.

In addition to Equations (1) and (2), two additional constraints on cycle‐consistency are imposed: 1) a structural similarity index measure (SSIM) loss; and 2) a topology‐preserving loss (centrelineDice or clDice)^[^ ^45^ ^]^ for backward cycle‐consistency. SSIM was used for forward‐consistency to ensure structural and perceptive features in biomedical images were retained when generating fake images. Similarly, to preserve the morphological characteristics of vascular networks when segmenting blood vessels, a constraint on backward‐consistency was applied that seeks to minimize differences in network structure and topology.

SSIM was a perceptually motivated metric composed of three comparison functions: luminance, contrast, and structure. Typically SSIM was used to assess image quality, so as a loss function could be used for image restoration,^[^ ^68^ ^]^ such as denoising and super‐resolution^[^ ^69^, ^70^ ^]^ and pose‐guided person image generation.^[^ ^71^ ^]^ Here, SSIM adopted a sliding Gaussian window to calculate the SSIM index between two local patches centred around a pixel coordinate. For image patches *I*_*p*_ and *J*_*p*_, SSIM is defined as

(3) $$\mathrm{SSIM}\left(I_{p},J_{p}\right)=\frac{\left(2\mu_{I}\mu_{J}+C_{1}\right)\left(2\sigma_{IJ}+C_{2}\right)}{\left(\mu_{I}^{2}+\mu_{J}^{2}+C_{1}\right)\left(\sigma_{I}^{2}+\sigma_{J}^{2}+C_{2}\right)}$$

here, µ_*I*_, µ_*J*_, $\sigma_{I}^{2}$, and $\sigma_{J}^{2}$ are the mean and variance of *I*_*p*_ and *J*_*p*_, respectively, σ_*IJ*_ is the covariance of *I*_*p*_ and *J*_*p*_, and *C*_1_ = (0.01 · *L*)^2^ and *C*_2_ = (0.03 · *L*)^2^, where *L* is the dynamic range of the pixel‐values. To maximize the SSIM of biomedical images, the reconstruction loss is formed:

(4) $$L_{rec}\left(G,F,x,y\right)=1-E_{x∼p_{data}\left(x\right)}\mathrm{SSIM}\left(x_{p},F{\left(G\left(x\right)\right)}_{p}\right)$$

where the subscript *p* indicates image patches.

Cycle‐consistency alone did not provide sufficient spatial constraint on the network topology of segmented images.^[^ ^45^ ^]^ Consequently, spatial and topological constraints on backward‐consistency were added to act as additional regulatory loss function term. Here, the segmentation labels of synthetically‐generated 3D vascular networks, *y*, were compared to *G*(*F*(*y*)) to ensure differences in topology were minimized. Minimization was achieved using the connectivity‐preserving metric **clDice**, which enforces topology preservation up to homotopy equivalence for binary segmentation.^[^ ^45^ ^]^ The loss function was a combination of soft‐Dice loss and the differentiable form of **clDice**, *soft* **clDice**:

(5) $$\begin{array}{cc} L_{topo}\left(G,F,y\right) & =E_{y∼p_{data}\left(y\right)}\left\{\left(1-\alpha \right)\left[1-soft\mathrm{Dice}\left(y,G(F(y))\right)\right]\right.\\ & \left.+\alpha \left[1-soft\mathrm{clDice}\left(y,G(F(y))\right)\right]\right\} \end{array}$$

where the weighting, α, is set to 0.5.

The adversarial loss was expressed via least‐squares adversarial loss^[^ ^72^ ^]^ to mitigate problems with vanishing gradients. In the case of the mapping *G*: *X* → *Y*, this is given by

(6) $$\begin{array}{ccc} L_{GAN}\left(F,D_{X},x,y\right) & = & \frac{1}{2}E_{y∼p_{data}\left(y\right)}\left[{\left(D_{X}\left(x+\varepsilon_{Y}\right)-1\right)}^{2}\right]\\ & & +\frac{1}{2}E_{x∼p_{data}\left(x\right)}\left[{\left(D_{X}\left(F\left(y\right)+\varepsilon_{X}\right)\right)}^{2}\right] \end{array}$$

where ϵ_*X*_ and ϵ_*Y*_ are the randomly sampled Gaussian noise.^[^ ^47^ ^]^ Here, the discriminator *D*_*X*_ aims to discern translated images, *F*(*y*), from real images *y* by minimizing the objective function, whereas *F* aims to maximize it against its adversarial rival. Adversarial loss was similarly used for the mapping *G*: *X* → *Y*. An identity mapping loss was not imposed, as this constrains the tint of an image^[^ ^42^ ^]^ and so was not required for the grayscale and binary image volumes.^[^ ^21^ ^]^

Thus, the objective function of VAN‐GAN is given by

(7) $$\begin{array}{c} \begin{array}{cc} L(G,F,D_{X},D_{Y}) & =L_{GAN}\left(G,D_{Y},x,y\right)+L_{GAN}\left(F,D_{X},x,y\right)\\ & +\lambda \left[L_{cyc}^{G}\left(G,F,x\right)+L_{cyc}^{F}\left(G,F,y\right)\right]\\ & +\eta \left[L_{rec}\left(G,F,x\right)+L_{topo}\left(G,F,y\right)\right] \end{array} \end{array}$$

where the hyperparameters λ and η control the relative importance between the objectives. The aim is to solve:

(8) $$G^{*},F^{*}=\arg\min_{G,F}\max_{D_{X},D_{Y}}L\left(G,F,D_{X},D_{Y}\right)$$

### Generating Synthetic Vasculature

A language‐theoretic model, called a Lindenmayer system (L‐System),^[^ ^73^ ^]^ was used to generate 3D branching vascular networks (termed here as *V‐System*). L‐Systems were ideally suited to the segmentation task as these models had been shown to create realistic, computer‐generated 3D vascular branching structures^[^ ^74^, ^75^ ^]^ quickly and at scale^[^ ^38^ ^]^ (O(10^2^) networks in O(1) minutes). To generate a synthetic branching network, a new stochastic grammar was used to create a string, which defines the complexity (in this case, the number of branching orders) of the vascular network, for example, branching order and angle, vessel diameter and tortuosity, and aneurysms or branching vessel shrinkage (see Supporting Information for mathematical descriptions). These strings were translated to graph form using a lexical and syntactic analyzer and subsequently converted into volumetric binary segmentation masks,^[^ ^38^ ^]^ forming a synthetic dataset of 459 images for network training.

### Performing Physics‐Driven Photoacoustic Simulations

To generate a paired image dataset, photoacoustic simulations were performed on the segmentation volume of each image in the synthetic vascular dataset. Each image pair consisted of a physics‐driven image volume and its corresponding known segmentation labels. Simulations followed the method of ref. ^[^ ^38^ ^]^ who used SIMPA^[^ ^76^ ^]^ (v0.1.1 with MCX v2020, 1.8) with the k‐Wave MATLAB toolbox^[^ ^77^ ^]^ (v1.3, MATLAB v2020b, MathWorks, Natick, MA, USA) to predict photoacoustic signals across synthetic vasculatures under the assumption that they were embedded in muscular tissue. In brief, vascular planar (XY) illumination was achieved on an isotropic resolution with optical forward modelling assuming an absorption spectrum of 50% oxygenated haemoglobin in blood vessels to mimic tumours.^[^ ^78^ ^]^ 3D acoustic forward modelling was then performed with the signal detected by a planar array of sensors positioned at the tissue surface, mimicking the PAI instrument (see below). The resulting photoacoustic wave‐field was then reconstructed using a fast Fourier transform.^[^ ^77^ ^]^ While the PAI instrument raster‐scans, this process was approximated with a planar illumination due to computational restrictions (reducing simulation time by a factor of 600^2^).

### Experimental Imaging

PAI was performed using a commercial system (Raster‐scan optoacoustic mesoscopy RSOM Explorer P50, iThera Medical GmbH), as described previously.^[^ ^38^ ^]^ Briefly, string phantoms were composed in agar mixed with intralipid (both Merck, UK) to mimic tissue‐like scattering with red‐coloured synthetic fibres (Smilco, USA) embedded at three different depths. PAI data were acquired at 100% laser energy with a 2kHz repetition rate. All animal procedures were conducted in accordance with project (PE12C2B96) and personal licenses (I544913B4, IA70F0365) issued under the United Kingdom Animals (Scientific Procedures) Act, 1986 and approved locally by the Cancer Research UK Cambridge Institute Animal Welfare Ethical Review Board.

To generate in vivo vascular tumour models, breast PDX tumour fragments were cryopreserved in a freezing media consisting of heat‐inactivated foetal bovine serum (10500064, Gibco^TM^, Fisher Scientific, Göteborg Sweden) and 10% dimethyl sulfoxide (D2650, Merck). The fragments were then defrosted at 37 °C, washed with Dulbecco's Modified Eagle Medium (41965039, Gibco), mixed with matrigel (354262, Corning, NY, USA), and surgically implanted into the flank of 6–9 week‐old NOD scid gamma (NSG) mice (#005557, Jax Stock, Charles River, UK), following standard protocols.^[^ ^38^, ^51^ ^]^ The implantation involved one oestrogen receptor negative (ER‐, *n* = 6) PDX model and one oestrogen receptor positive (ER+, *n* = 8) PDX model. After the tumours had reached an average diameter of ≈1 cm, the mice were imaged and then sacrificed, with the tumours collected in formalin for IHC analysis.

For the remaining breast cancer cell lines, seven‐week old immuno‐deficient female nude (BALB/c nu/nu) mice (Charles River) were inoculated orthotopically in the mammary fat pad of both flanks 1 · 100^6^ cells (either MCF7, *n* = 7, or MDA‐MB‐231, *n* = 6, random group assignment) in a final volume of 100 µL of 1:1 phosphate‐buffered saline (PBS, Gibco) and matrigel (BD). For MCF7, oestrogen implants (E2‐M ‐ 127 β‐estradiol 90 days release, daily dose: 41.2‐105.6 pgml^‐1^, Belma Technologies) were implanted subcutanaously in the scruff of the neck three days before tumour cell injection.

For animal imaging, were mice anaesthetised using 3–5% isoflurane in 50% oxygen and 50% medical air. Mice were shaved and depilatory cream applied to remove hair that could generate image artefacts; single mice were placed into the PAI system, on a heat‐pad maintained at 37°C. Respiratory rate was maintained between 70–80 bpm using isoflurane (≈1 − 2% concentration) throughout image acquisition. PAI data were acquired at 80% laser energy at 1kHz.

For string phantom imaging, phantoms were prepared following standard protocols^[^ ^79^ ^]^ using agar mixed with intralipid (both Merck, UK) to mimic tissue‐like scattering with red‐colored synthetic fibres (Smilco, USA) embedded at three different depths (top: 0.5 mm, middle: 1 mm, bottom: 2 mm).

### Immunohistochemistry

The tumour tissues, obtained for ex vivo validation, were processed by sectioning formalin‐fixed paraffin‐embedded (FFPE) samples. After deparaffinisation and rehydration, IHC analysis was performed on the tissues using the following antibodies: CD31 (anti‐mouse 77699, Cell Signalling, London, UK), αSMA (anti‐mouse ab5694, Abcam, Cambridge, UK), and carbonic anhydrase‐IX (CAIX) (anti‐human AB1001, Bioscience Slovakia, Bratislava, Slovakia), at concentrations of 1:100, 1:500, and 1:1000, respectively. The analysis was carried out using a BOND automated stainer, with a bond polymer refine detection kit (Leica Biosystems, Milton Keynes, UK), and 3,3'‐diaminobenzadine as a substrate. The stained FFPE sections were scanned at a magnification of 20x using an Aperio ScanScope (Leica Biosystems, Milton Keynes, UK) and analyzed with either ImageScope software or HALO Software (v2.2.1870, Indica Labs, Albuquerque, NM, USA). Regions of interest (ROIs) were drawn over the entire viable tumour area, and the built‐in algorithms were customized to analyze the following: CD31 positive area (µm^2^) normalized to the ROI area (µm^2^) (reported as CD31 positivity (%)), area of CD31 positive pixels (µm^2^) colocalized on adjacent serial section with αSMA positive pixels/CD31 positive area (µm^2^).

### Datasets and Image Preprocessing

Image volume datasets were split into two categories: synthetic or experimental datasets (see Table S6, Supporting Information for overview). The synthetic datasets were comprised of binary segmentation labels of 3D mathematically‐generated vascular networks, which were paired with computer‐simulated photoacoustic image volumes (*n* = 449 for each). Here, physics‐driven predictions were performed on the spatial architecture of the synthetic vasculature in each binary image volume provided by the V‐System. The experimental datasets consisted of a string phantom (*n* = 7), mouse ears (*n* = 32), mouse skin (*n* = 41), breast cancer patient‐derived xenograft (PDX) tumours in mice (*n* = 445), MCF7 and MDA‐MB‐231 tumours derived from bread cancer cell lines (*n* = 204) and human breast angiograms (*n* = 2, acquired by an independent lab using 3D‐PACT),^[^ ^52^ ^]^ all imaged in vivo. These datasets represent the imaging domain in VAN‐GAN where *n* indicates the number of image volumes used. Note, the string phantoms and human breast angiograms were not used for training.

All RSOM datasets were stored as either 32‐bit grayscale 600 × 600 × 140µm^3^ (experimental data) or 512 × 512 × 140µm^3^ (simulated data) single channel tiff stacks with an isotropic voxel size of 20 × 20 × 20µm^3^ in (X,Y,Z) coordinates, where the Z‐axis is perpendicular to the surface. All synthetic segmentation images were stored in an eight‐bit format and generated with dimensions 512 × 512 × 140µm^3^ and an equal isotropic voxel size. As VAN‐GAN trains on image subvolumes of size 128 × 128 × 128 voxels, all datasets were downsampled to 128 voxels in the Z‐axis using bicubic downsampling, to ensure that depth‐dependent SNR information was retained for training. All real and simulated photoacoustic images were normalized by performing XY slice‐wise Z‐score normalisation followed by thresholding of the top and bottom 0.05% of pixel intensities to correct for uneven illumination with respect to depth. Finally, datasets were normalized to a pixel intensity range of [− 1, 1]. Datasets were partitioned with 10% assigned for testing and with the remaining 90% split 80/20 between training/validation.

Photoacoustic images acquired by 3D‐PACT were store as 32‐bit grayscale single channel tiff stacks with an isotropic voxel size of 50 × 50 × 50µm^3^. The raw images supplied open‐source^[^ ^52^ ^]^ were segmented without normalization.

### Network Training

Image patches were randomly sampled with a global batch size of two. Given the predominance of background over vessel structures, a special mapping function was employed when retrieving images from the synthetic segmentation dataset. This function was crucial in guiding the VAN‐GAN model to concentrate on vessel segmentation rather than merely identifying the expansive background areas. Specifically, the function checked each sampled volume for the presence of any vessel structures. For 90% of sampled volumes, if no vessel was detected, a new image volume was sampled. In addition, to artificially extend the size of the datasets, all images were augmented via a rotation about the Z‐axis randomly sampled from the set {0,π/2,π,3π/2,2π}.

All convolutional kernels were initialized using a He‐normal initialiser and loss functions hyperparameters were set to default values^[^ ^42^, ^45^ ^]^ α = 0.5, λ = 10 and η = 5. Following,^[^ ^42^ ^]^ training was performed for 200 epochs using the Adam optimizer^[^ ^80^ ^]^ with a learning rate of 2 × 10^−4^ and 1^st^ and 2^nd^ moment estimates of 0.5 and 0.9, for both generators and discriminators. Each generator and discriminator was given a linear learning rate decay to 0 from the 100^th^ epoch.

Training was stabilized in two ways. First, noise was applied to the real and fake input images to the discriminators.^[^ ^47^ ^]^ Noise was sampled from a Gaussian distribution with a mean and annealed variance of 0.0 and 0.1(1 − *i*/200), where *i* was the i^th^ epoch. Second, to avoid exploding gradients, a gradient clip normalisation strategy^[^ ^81^ ^]^ where all gradients of each weight was individually clipped so that its norm is no higher than 100.

### Image Postprocessing

To reconstruct whole image volumes from generator output a sliding‐window approach was employed.^[^ ^34^ ^]^ A window of size 128 × 128 × 128 voxels was employed to traverse each input image, moving with a stride of 25 voxels in both the X and Y directions. For each voxel location, the intensity values within the window were summed. The mean intensity value was then computed by tracking the frequency with which the window covered each voxel during the process. To reduce edge artefacts, symmetric padding was used to ensure the window passed over each voxel for an equal number of instances. This method results in a 32‐bit 3D grayscale image where intensities indicate a probability that a voxel was a blood vessel. After applying bicubic upsampling to achieve isotropic voxel dimensions (specifically, 140 voxels along the Z‐axis), the images were then thresholded using the histogram of voxel intensities to binarise the image.

### Evaluation Metrics

Common machine learning metrics did not provide a complete picture of image segmentation performance for tubular‐like structures.^[^ ^82^ ^]^ To evaluate the results, both standard segmentation metrics and a set of vascular descriptors were calculated^[^ ^38^, ^83^ ^]^ to provide a deeper insight into how well network morphology was predicted. The standard segmentation metrics used comprised of: F1 Score = 2 · (*precision* · *sensitivity*)/(*precision* + *sensitivity*); Intersection over Union (IoU) = *TP*/(*TP* + *FP* + *FN*); Sensitivity = *TP*/(*TP* + *FN*) and Specificity = *TN*/(*TN* + *FP*), where *TP* = true positive, *TN* = true negative, *FP* = false positive and *FN* = false negative.

To calculate vascular descriptors, all segmentations were skeletonised using the open‐source package Russ‐learn.^[^ ^84^, ^85^ ^]^ The vascular skeletons allowed to perform structural and topological data analyses on the vascular skeletons.^[^ ^83^, ^86^ ^]^ The metrics used in this study were: standard deviation of vessel diameters, network volume, surface density (the surface area of the vascular network normalised against the tissue volume), connected components (or subnetworks, Betti‐0), looping structures (Betti‐1), and network connectivity (the volume of the largest vascular subnetwork normalized against total network volume).

### Statistical Analysis

Statistical analyses were performed using Prism software (version 9, GraphPad Software, San Diego, CA, USA). A variety of statistical tests were tailored to the specific conditions of the data sets to ensure robustness and reliability of the results. For the comparison of synthetic segmentations, the non‐parametric Friedman test was employed, which was suitable for datasets that might not follow a normal distribution. In the analysis of in vivo ear datasets, vascular descriptors were examined using either parametric t‐tests or non‐parametric Wilcoxon signed‐rank tests, depending on whether the data met normality assumptions. For in vivo tumour datasets, inter‐group comparisons between different tumour types were conducted using the Mann‐Whitney U tests.

To ensure consistent and objective outlier management, statistical outliers were identified through a combination of non‐parametric methods including Tukey's fences, Median Absolute Deviation (MAD), the Modified Z‐Score method, and percentile cutoffs at the 5^th^ and 95^th^ percentiles, along with the Hampel identifier. All tests were conducted as two‐sided with a significance threshold set at a *P*‐value of <0.05.

## Conflict of Interest

The authors declare no conflict of interest.

## Author Contributions

Conceptualisation is performed by P.W.S. and S.E.B. Methodology is performed by P.W.S. and J.G.. Software is provided by P.W.S.. Validation is done by P.W.S. and S.E.B.. Formal Analysis is performed by P.W.S.. Investigation is done by P.W.S. and L.H.. Resources are provide by S.E.B.. Data Collection is performed by P.W.S., L.H., T.L.L., and E.L.B.. Data Curation is performed by P.W.S.. Writing ‐ original draft is completed by P.W.S.. Writing ‐ review & editing is perdormed by P.W.S., L.H., T.L.L., E.L.B., J.G., and S.E.B.. Visualisation is performed by P.W.S.. Project Administration is done by P.W.S. and S.E.B.. Funding Acquisition is provided by P.W.S. and S.E.B..

## Supporting information

Supporting Information

## Data Availability

The data that support the findings of this study are openly available in VAN‐GAN Data Repository at https://doi.org/10.17863/CAM.96379 , reference number 96379.
