# Supplementary material for: Unsupervised Segmentation of 3D Microvascular Photoacoustic Images Using Deep Generative Learning
Source: Adv Sci (Weinh). 2024 Jun 23;11(32):2402195. doi: 10.1002/advs.202402195 (PMC11348209; doi:10.1002/advs.202402195)
Supplement: Supplementary file 1 — Supporting Information [file ADVS-11-2402195-s001.pdf]

ADVANCED SCIENCE
Open Access

Supporting Information

for *Adv. Sci.*, DOI 10.1002/advs.202402195

Unsupervised Segmentation of 3D Microvascular Photoacoustic Images Using Deep Generative Learning

Paul W. Sweeney, Lina Hacker, Thierry L. Lefebvre, Emma L. Brown, Janek Gröhl and Sarah E. Bohndiek**

Supplementary Material: Unsupervised Segmentation of 3D Microvascular Photoacoustic Images Using Deep Generative Learning

Paul W. Sweeney Lina Hacker Thierry L. Lefebvre Emma L. Brown Janek Gröhl Sarah E. Bohndiek**

Dr P. W. Sweeney, Dr L. Hacker, T. L. Lefebvre, Dr E. L. Brown, Dr J. Gröhl, Prof. S. E. Bohndiek
Cancer Research UK Cambridge Institute, University of Cambridge, Robinson Way, Cambridge, CB2 0RE, UK
Email Address: paul.sweeney@cruk.cam.ac.uk; seb53@cam.ac.uk

Dr P. W. Sweeney, Dr L. Hacker, T. L. Lefebvre, Dr E. L. Brown, Dr J. Gröhl, Prof. S. E. Bohndiek
Department of Physics, University of Cambridge, JJ Thomson Avenue, Cambridge, CB3 0HE, UK

Keywords: *Unsupervised, deep learning, generative, segmentation, blood vessels, photoacoustics*

1 Supplementary Notes

1.1 Imaging Artefacts in Photoacoustic Mesoscopy

Photoacoustic mesoscopy is a non-invasive imaging technique that uses optical excitation and acoustic detection to produce high-resolution images of biological tissues. However, the technique is susceptible to imaging artefacts that can distort images and reduce image quality.[1] These artefacts arise from acoustic attenuation, acoustic reflection, optical scattering, acoustic diffraction, and thermal effects. Illumination artefacts, arise due to the limited top illumination of the field of view, leading to optical excitation and acoustic wave generation only in the upper part of absorbing structures that face the illumination and transducer array, resulting in inaccurate diameter estimations in the XZ plane compared to the XY plane.[2] Shadow artefacts, arise from obscuring objects, causing a signal loss in underlying objects due to strong optical or acoustic attenuation of the overlaying structure. Reflection artefacts, occur due to presence of acoustic reflectors/scatterers or strong acoustic reverberations of the absorbing object itself,[3–5] leading to a signal echo near the object of interest. They can occur in-plane or out-of-plane depending on the position of the absorber/reflector.[6]

1.2 A Comparison of Segmentation Methods for Tumour Vasculature

A comparison of performance between RF and VAN-GAN was performed for our PDX dataset for comparison to the results of [2] (Figure S7A-C). Results were consist with prior analyses where VAN-GAN prediction significantly larger vascular density ($P < 0.0001$, Figure S7D) and vessel diameters ($P < 0.01$, Figure S7E). Vasculature segmented by VAN-GAN also exhibited greater connectivity compared to RF ($P < 0.001$, Figure S7F) resulting in a significant difference in computed vessel looping structures ($P < 0.001$, Figure S7G).

VAN-GAN allowed us to distinguish between structural differences between the ER+ and ER- subtypes, where ER+ tumours exhibited significantly larger mean blood vessel lengths ($P < 0.01$), which was not

statistically significant using RF in [2] (Figure S8A). Similarly to [2], VAN-GAN predicts a significant difference in mean vessel diameters between the subtypes (Figure S8B), however, the trend is reversed with ER+ tumours on average exhibiting significantly larger diameters compared to ER- ($P < 0.01$). This highlights how VAN-GAN can provide an unbiased insight into biological characteristics of solid tumours.

2 Supplementary Methods

2.1 V-System Stochastic Grammar

The aim of VAN-GAN is to learn the mappings between imaging and segmentation domains. Whilst datasets containing image volumes of real vascular networks are readily available, 3D blood vessel segmentations are not creating a need for realistic, 3D blood vessel segmentation labels for VAN-GAN to learn from. Mathematical angiogenesis models exist which incorporate biological mechanisms to simulate realistic, interconnected blood vessel networks,[7] however, to generate a large library of vascular networks of a size applicable to deep learning can be computationally expensive.

Our synthetic vascular networks are generated using L-System stochastic definitions (termed here, *V-System*) that define a rule as *name* → *successor*, where symbols are replaced according to the respective rule.[8,9] Following [9], rules are composed by symbols that have the following actions:

- $f(l, d)$: forward l units in the direction vector and record vessel diameter, d;
- $+(\theta)$ and $-(\theta)$: rotate direction vector clockwise or anti-clockwise by θ° on the plane defined by the current direction vector;
- $/(\phi)$: rotate the current plane by ϕ° about the direction vector;
- *[*: store the current state formed by the diameter;
- *]*: restores the last saved state and allows the generation of a new branch;
- { and }: define the limits of a vessel segment.

For increasing number of recursive iterations, n, the string of instructions progressively becomes more complex. For example, in the case of modelling the growth of algae,[8] using the grammatical rules A→ AB and A→ B, the axiom A becomes:

$n = 0$: A

$n = 1$: AB

$n = 2$: ABA

$n = 3$: ABAAB

$n = 4$: ABAABABA

The following string grammar was used to generate our synthetic vascular trees:

$$F \rightarrow S(n-1, d_0)[+(\theta_1)/(\phi_1)F(n-1, \theta_1)][-(\theta_2)/(\phi_2)F(n-1, d_2)], \quad (1)$$

where d_1 and d_2, and θ_1 and θ_2 are daughter branching diameters and bifurcation angles, respectively, stochastically calculated from previous studies.[9–11] The vessel tilt angles, ϕ_1 and ϕ_2, are sampled from

a uniform distribution over the interval $[22.5°, 27.5°]$ multiplied by a random $\pm$ sign. Here, the string grammar S is defined by:

$$S(n, d_0, \alpha, \beta) \rightarrow \begin{cases} \{S_0(n, d_0)\} & \text{if} \quad r < 0.5 \\ \{S_1(n, d_0)\} & \text{if} \quad r \geq 0.5 \end{cases}, \tag{2}$$

where r is a random number generated over the interval $[0, 1)$. The function S_i for $i = 0, 1$ is defined by

$$S_i(n, d_0) \rightarrow \begin{cases} D(n-1, \lambda_1 d_0) \pm (\theta_1) D(n-1, \lambda_2 d_0) & \\ \mp(\theta_2) D(n-1, \lambda_3 d_0) \mp (\theta_1) D(n-1, \delta_1 d_0) & \\ \pm(\theta_2) D(n-1, \delta_2 d_0) & \text{if} \quad r < 0.1 \\ D(n-1, d_0) \pm (\theta_1) D(n-1, d_0) \mp (\theta_2) D(n-1, d_0) & \\ \mp(\theta_1) D(n-1, d_0) \pm (\theta_2) D(n-1, d_0) & \text{if} \quad r \geq 0.1 \end{cases}, \tag{3}$$

where λ_j for $j = 1, 2, 3$ and δ_k for $k = 1, 2$ are growth and decay rates, respectively, sampled from the interval $[1, 1.5]$. We note that $\boldsymbol{\lambda}$ and $\boldsymbol{\delta}$ are vectors sorted in ascending and descending orders of magnitude, respectively. The string S_i defines a branching vessel, which can randomly contain an aneurysm of random size (probability of 1 in 10).

$$D(n, d_0) \rightarrow f(L(d_0), d_0), \tag{4}$$

where $L(d_0)$ the segment length function which randomly samples a value from the interval $[\epsilon d_0/5 - \Delta, \epsilon d_0/5 + \Delta]$. Here, the division by five is due to (3) where vessels are subdivided into five segments, and ϵ and Δ are randomly sampled from the intervals $[4, 10]$ and $[2, 4]$, respectively.

Using our grammatical rule, as an example, the axiom F becomes:

$n = 0$: A

$n = 1$: {S}[+(38.3)/(0.0)F][-(36.7)/(0.0)F]

$n = 2$: {D+(0.0)D-(0.0)D-(0.0)D+(0.0)D}[+(37.4)/(25.5){S}[+(51.4)/(0.0)F][-(24.2)/(0.0)F]][-(37.6)/(-25.7){S}[+(24.6)/(-24.0)F][-(51.0)/(-22.6)F]].

The resulting synthetic vascular network quantified for a set of statistical and topological vascular descriptors[2]. Exemplar vascular skeletons of increasing complexity are illustrated in Figure S1 with distributions for our metrics given in Figure S2.

2.2 Supervised Random Forest (RF) Pixel Classifier

Using the open source ilastik tool, users can pre-define a set of possible image features and manually annotate a subset of voxels in training images (either 2D or 3D images) for a random forest model to learn to recognise complex image features. The trained model can then be applied to the remaining unlabelled data and its accuracy improved through retrospective interactive training. Photoacoustic images were preprocessed for application to ilastik's random forest toolbox by applying a slice-wise median filter with a kernel size of 3×3 to remove noise and improve contrast. For each dataset, a sample of images were manually annotated with two label categories: blood vessel and tissue background. Here, approximately 10 XY images were labelled from each training image volume. All 3D colour / intensity, edge and texture features were selected for all Gaussian mean variational scales (0.3 to 10.0) to extract fine details at a small scale as well features from larger voxel neighbourhoods. RF models were trained by authors PWS, LH, TLL and ELB. For synthetic data, all vessel labels were provided for training along with a proportion of surrounding background labels.[2] Trained models were applied to unlabelled images with predictions thresholded using default settings to create binary segmentation masks.

2.3 Model Training

VAN-GAN models were trained pragmatically as and when new datasets and computational hardware became available. Three VAN-GAN models were trained in total (see Table S7). In each case, VAN-GAN predictions were thresholded at an intensity value of 0.5 (images scaled between 0 and 1.0) to create a segmentation mask. Increasing the batch size led to a noticeable improvement in VAN-GAN certainty between the vessel and tissue background. We observed that workstation (2), which employed a larger batch size of 4 (global batch size of 16), achieved better results compared to workstation (1) that used a batch size of 1 (global batch size of 2).

2.4 Comparing Vessel Diameters between Axial Planes

To compare the diameters of vessels in the axial XY- and XZ-planes, analysis was performed using MATLAB v2022b, MathWorks, Natick, MA, USA. Maximal intensity projections (MIPs) were created in each axial plane for each image. The diameters were analysed by creating a distance transform (using in-built function *bwdist*) to provide vessel diameters and vascular skeletons (using in-built function *bwmorph*). The resulting data provided a means to calculate mean vessel diameter for each plane. We note that MIPs were used as skeletonisation assumes radial symmetry, however, accuracy of this method declines for increasing vascular density of an image due to overlapping vessels when generating MIPs.

3 Supplementary Figures

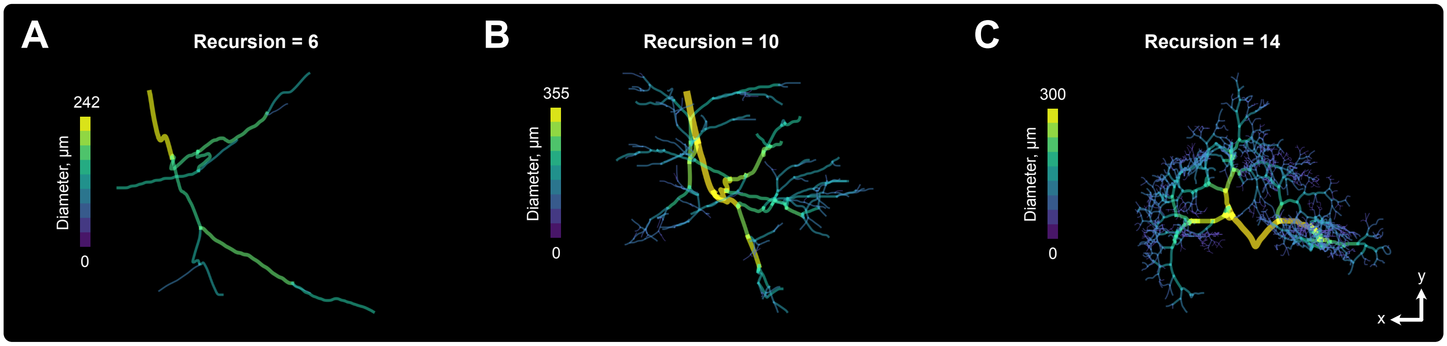

Figure S1: **Exemplar synthetic vasculatures of varying complexity.** 2D projections of networks, colour-coded to vessel diameters, are provided for increasing network complexity for (A) eight, (B) eleven and (C) fourteen grammatical recursions.

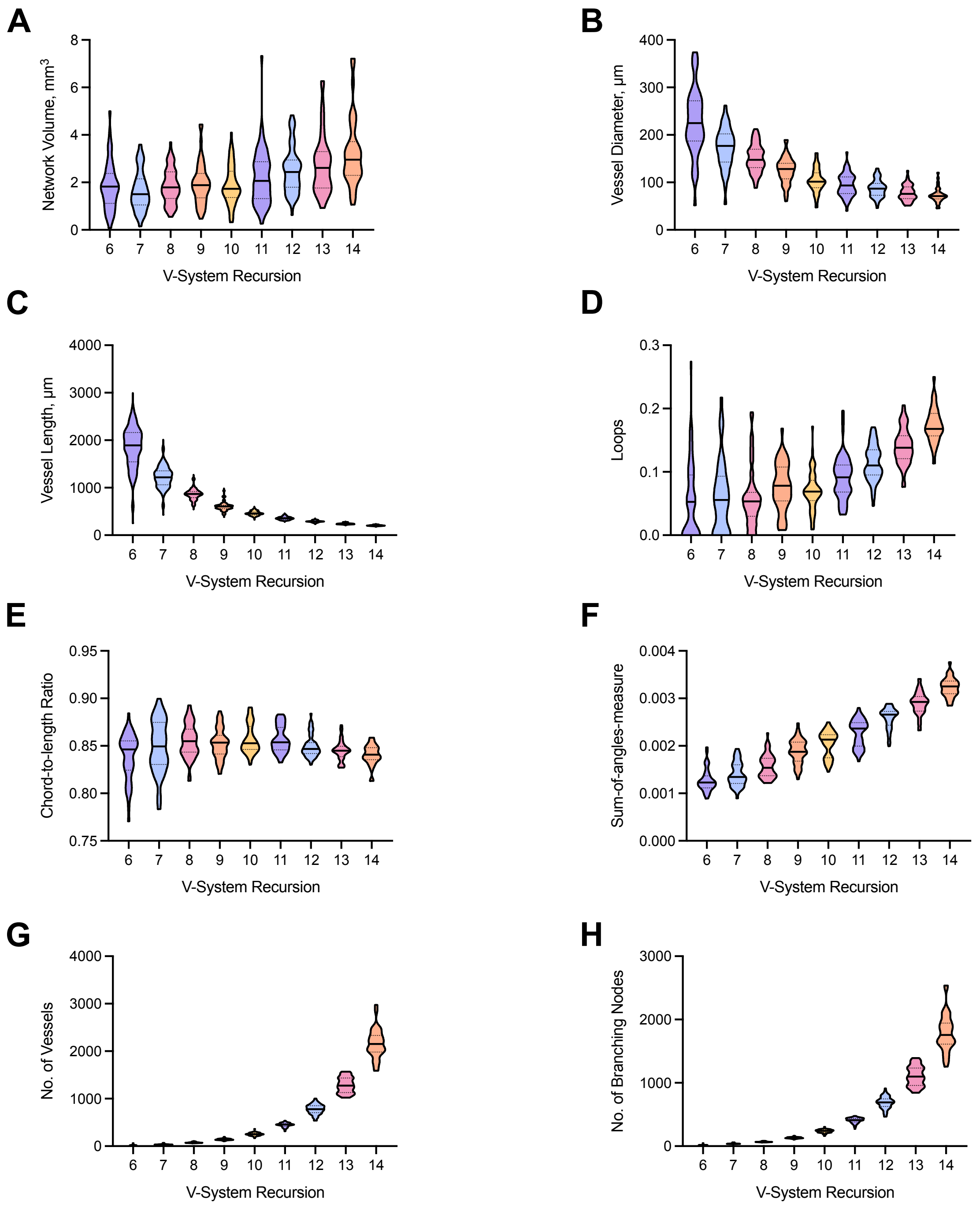

Figure S2: **Statistical and vascular descriptor distributions calculated from the ground truth synthetic vascular networks.** Truncated violin plots are separated into network complexity indicated by the number of V-System recursions. (A) Network volume, (B) mean vessel diameters, (C) mean vessel lengths, (D) mean number of network loops, (E) chord-to-length (CLR) ratio which defines vessel curvature, (F) sum-of-angles-measure (SOAM, a measure of vessel tortuosity), and the total number of (G) vessels and (H) branching nodes. Dashed and dotted lines indicate median, and upper (75th) and lower (25th) quartiles, respectively. Note, connected components and loops are normalised with respect to total number of edges per network.

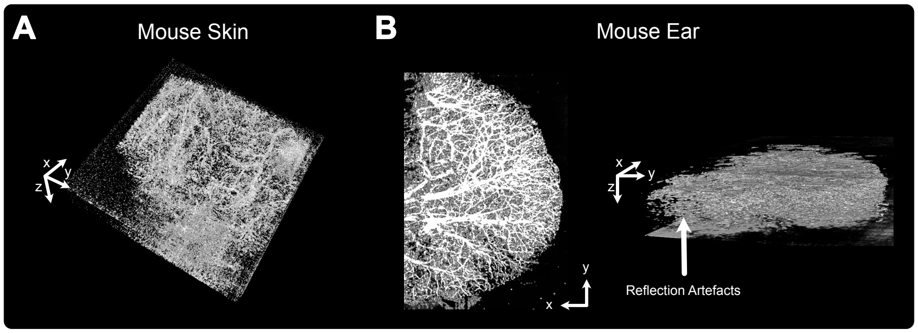

Figure S3: **Poor trained U-Net generalisability to *in vivo* data.** Application of our U-Net model trained on our simulated paired data to *in vivo* photoacoustic images of (A) mouse skin and (B) mouse ear. The U-Net model over segmented background noise such as reflection artefacts (shown in (B)).

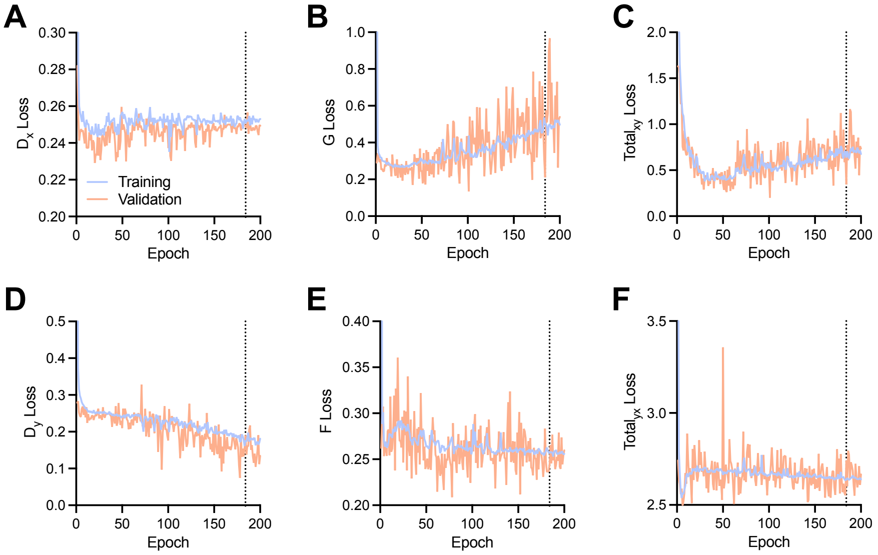

Figure S4: **VAN-GAN training and validation losses when applied to synthetic imaging and segmentation domains.** Training and validation losses are given per epoch for: discriminators (A) D_x and (D) D_y; generators (B) G and (E) F; and total losses (C) $x \rightarrow y$ and (F) $y \rightarrow x$. Dashed lines indicate the optimal epoch (epoch 184).

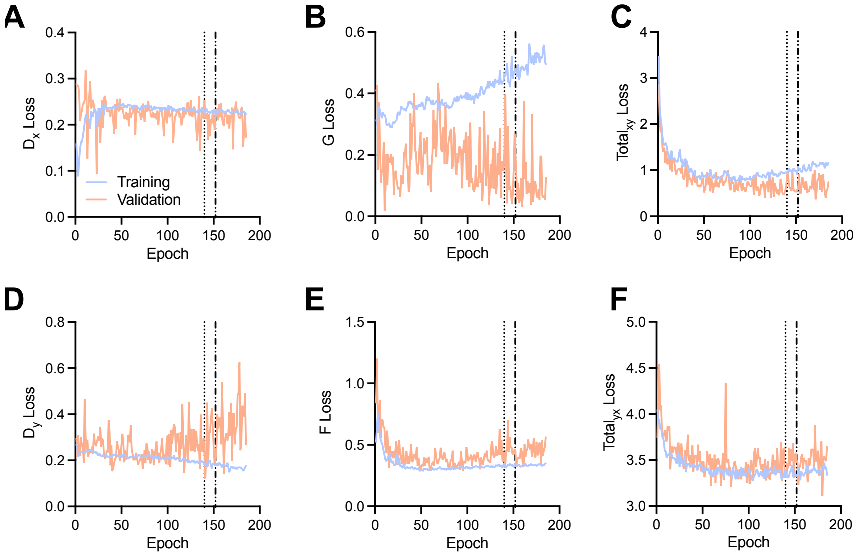

Figure S5: **VAN-GAN training and validation losses when applied to *in vivo* photoacoustic images of mouse skin, ear and breast cancer patient-derived xenograft tumours.** Training and validation losses are given per epoch for: discriminators (A) D_x and (D) D_y; generators (B) G and (E) F; and total losses (C) $x \rightarrow y$ and (F) $y \rightarrow x$. Dotted lines indicate epoch (140) used to segmented the ear dataset. Dashed lines indicate the optimal epoch (epoch 152). VAN-GAN experienced a vanishing gradient at the 185th epoch.

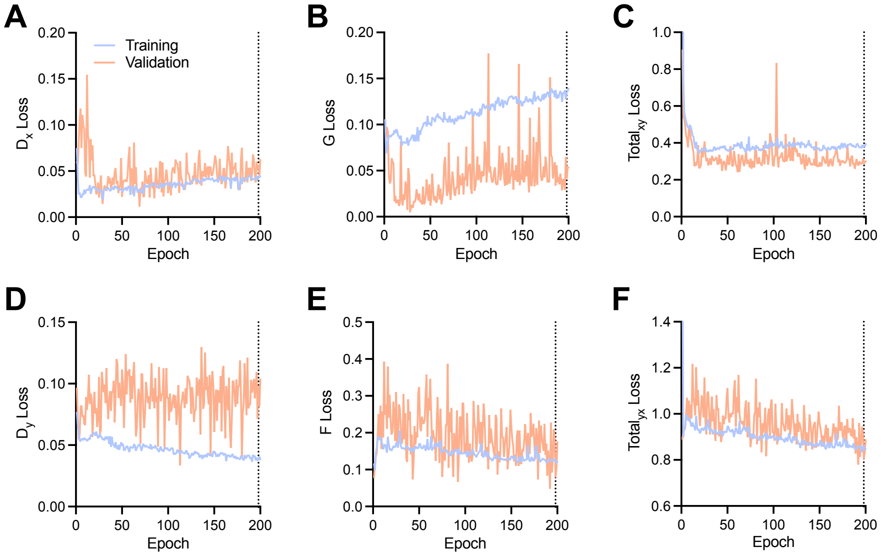

Figure S6: **VAN-GAN training and validation losses when applied to *in vivo* photoacoustic images of mouse skin, ear, breast cancer patient-derived xenograft tumours, and MCF7 and MDA-MB-231 tumours.** Training and validation losses are given per epoch for: discriminators (A) D_x and (D) D_y; generators (B) G and (E) F; and total losses (C) $x \rightarrow y$ and (F) $y \rightarrow x$. Dotted lines indicate epoch (140) used to segmented the ear dataset. Dashed lines indicate the optimal epoch (epoch 196).

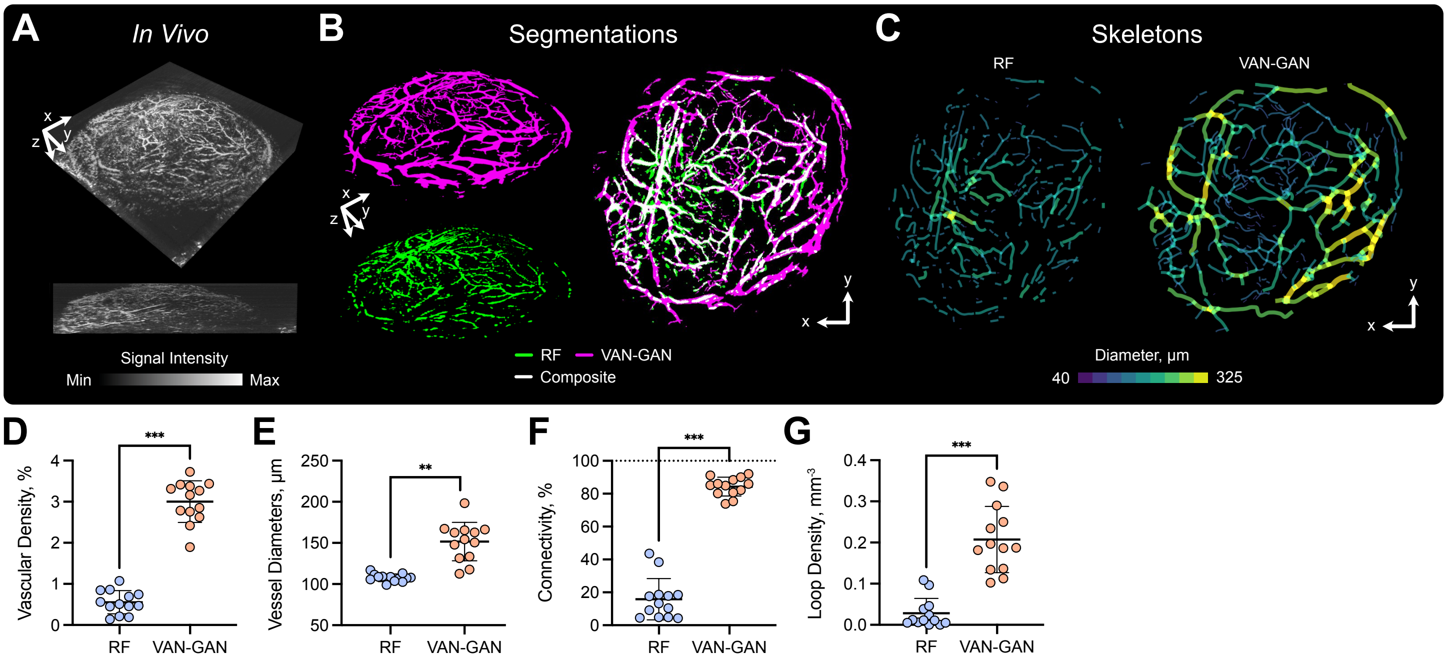

Figure S7: **VAN-GAN improves quantification of pathological vascular architecture.** Maximal intensity projections of: (A) *in vivo* signal intensity, (B) segmentation masks for (top left) VAN-GAN and (bottom left) the random forest pixel classifier (RF), and (right) a 2D overlay; and (C) vascular skeletons computed from (left) RF and (right) VAN-GAN segmentation predictions. We compute our vascular descriptors to compare results between the RF and VAN-GAN models. (D) Vascular density (the percentage of vascular volume with respect to tumour volume, %). (E) Mean vessel diameters (μm). (F) Connectivity (the volume percentage of the largest vascular subnetwork with respect to the sum of all subnetwork volumes, %). (G) Loop density (the number of network loops normalised against tumour volume, mm^{-3}). Mean and standard deviation of data are shown. Statistical significance indicated by * ($P < 0.05$), ** ($P < 0.01$), *** ($P < 0.001$) and **** ($P < 0.0001$).

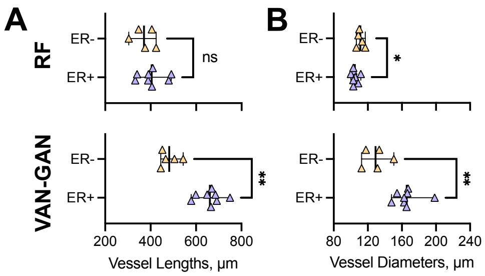

Figure S8: **Segmentation method can influence biological conclusions.** Computed mean vessel (A) lengths and (B) diameters for random forest classifier (RF, top) and VAN-GAN (bottom). Mean and standard deviation of data are shown. Statistical significance indicated by * ($P < 0.05$) and ** ($P < 0.01$) (ns = not significant).

4 Supplementary Tables

Table S1: **Network architecture for 3D deep residual U-Net.** Image input and output sizes are $128 \times 128 \times 128 \times 1$ (depth $\times$width $\times$height $\times$channel). *Conv i* represents the ith 3D convolutional layer. Note, for each encoding and decoding layer an identity mapping also exists in each residual block. Here, a 3D convolution of size $1 \times 1 \times 1$ is applied to the layer input and added to the layer output. The number of filters are equal to the other filters defined in each layer, and stride lengths are equal to 2 and 1 for encoding and decoding paths, respectively.

	Level	Layer	Filters	Size	Stride	Output Size
Input						$128 \times 128 \times 128 \times 1$
Encoder	1	Conv 1	16	$3 \times 3 \times 3$	1	$128 \times 128 \times 128 \times 16$
		Conv 2	16	$3 \times 3 \times 3$	1	$128 \times 128 \times 128 \times 16$
	2	Conv 3	32	$3 \times 3 \times 3$	2	$64 \times 64 \times 64 \times 32$
		Conv 4	32	$3 \times 3 \times 3$	1	$64 \times 64 \times 64 \times 32$
	3	Conv 5	64	$3 \times 3 \times 3$	2	$32 \times 32 \times 32 \times 64$
		Conv 6	64	$3 \times 3 \times 3$	1	$32 \times 32 \times 32 \times 64$
	4	Conv 7	128	$3 \times 3 \times 3$	2	$16 \times 16 \times 16 \times 128$
		Conv 8	128	$3 \times 3 \times 3$	1	$16 \times 16 \times 16 \times 128$
Bridge	5	Conv 9	256	$3 \times 3 \times 3$	1	$8 \times 8 \times 8 \times 256$
		Conv 10	256	$3 \times 3 \times 3$	1	$8 \times 8 \times 8 \times 256$
		Conv 11	256	$3 \times 3 \times 3$	1	$8 \times 8 \times 8 \times 256$
		Conv 12	256	$3 \times 3 \times 3$	1	$8 \times 8 \times 8 \times 256$
Decoder	4	Upsample 1	-	$2 \times 2 \times 2$	-	$16 \times 16 \times 16 \times 256$
		Conv 13	128	$3 \times 3 \times 3$	1	$16 \times 16 \times 16 \times 128$
		Conv 14	128	$3 \times 3 \times 3$	1	$16 \times 16 \times 16 \times 128$
	3	Upsample 2	-	$2 \times 2 \times 2$	-	$32 \times 32 \times 32 \times 128$
		Conv 15	64	$3 \times 3 \times 3$	1	$32 \times 32 \times 32 \times 64$
		Conv 16	64	$3 \times 3 \times 3$	1	$32 \times 32 \times 32 \times 64$
	2	Upsample 3	-	$2 \times 2 \times 2$	-	$64 \times 64 \times 64 \times 64$
		Conv 17	32	$3 \times 3 \times 3$	1	$64 \times 64 \times 64 \times 32$
		Conv 18	32	$3 \times 3 \times 3$	1	$64 \times 64 \times 64 \times 32$
	1	Upsample 4	-	$2 \times 2 \times 2$	-	$128 \times 128 \times 128 \times 32$
		Conv 19	16	$3 \times 3 \times 3$	1	$128 \times 128 \times 128 \times 16$
		Conv 20	16	$3 \times 3 \times 3$	1	$128 \times 128 \times 128 \times 16$
Output		Conv 21	1	$1 \times 1 \times 1$	1	$128 \times 128 \times 128 \times 1$

Table S2: **Network architecture for 3D convolutional discriminator.** *Conv i* represents the ith 3D convolutional layer. Note, Gaussian noise was added prior to each convolution and spatial dropout subsequently applied post-convolution.

	Conv Layer	Filters	Kernel Size	Stride	Dropout	Output Size
Input						$128 \times 128 \times 128 \times 1$
	Conv 1	64	$4 \times 4 \times 4$	2	20%	$128 \times 128 \times 128 \times 64$
	Conv 2	128	$4 \times 4 \times 4$	2	20%	$64 \times 64 \times 64 \times 128$
	Conv 3	256	$4 \times 4 \times 4$	2	20%	$32 \times 32 \times 32 \times 256$
	Conv 4	512	$4 \times 4 \times 4$	2	20%	$16 \times 16 \times 16 \times 512$
Output	Conv 5	1	$3 \times 3 \times 3$	1	0%	$16 \times 16 \times 16 \times 1$

Table S3: **Network architecture for 3D U-Net.** Image input and output sizes are $128 \times 128 \times 128 \times 1$ (depth $\times$ width $\times$ height $\times$ channel). 3D convolutions and max pooling used.

	Level	Layer	Filters	Kernel Size	Stride	Output Size
Input						$128 \times 128 \times 128 \times 1$
Encoder	1	Conv 1	32	$3 \times 3 \times 3$	1	$128 \times 128 \times 128 \times 16$
		Max Pooling 1	-	$3 \times 3 \times 3$	2	$128 \times 128 \times 128 \times 16$
	2	Conv 3	64	$3 \times 3 \times 3$	1	$64 \times 64 \times 64 \times 32$
		Max Pooling 2	-	$3 \times 3 \times 3$	2	$64 \times 64 \times 64 \times 32$
	3	Conv 5	128	$3 \times 3 \times 3$	1	$32 \times 32 \times 32 \times 64$
		Max Pooling 3	-	$3 \times 3 \times 3$	2	$32 \times 32 \times 32 \times 64$
	4	Conv 7	256	$3 \times 3 \times 3$	1	$16 \times 16 \times 16 \times 128$
		Max Pooling 4	-	$3 \times 3 \times 3$	2	$16 \times 16 \times 16 \times 128$
Bridge	5	Conv 9	512	$3 \times 3 \times 3$	1	$8 \times 8 \times 8 \times 256$
		Conv 10	512	$3 \times 3 \times 3$	1	$8 \times 8 \times 8 \times 256$
Decoder	4	Upsample 1	-	$2 \times 2 \times 2$	-	$16 \times 16 \times 16 \times 128$
		Conv 11	256	$3 \times 3 \times 3$	1	$16 \times 16 \times 16 \times 128$
		Conv 12	256	$3 \times 3 \times 3$	1	$16 \times 16 \times 16 \times 128$
	3	Upsample 2	-	$2 \times 2 \times 2$	-	$32 \times 32 \times 32 \times 128$
		Conv 13	128	$3 \times 3 \times 3$	1	$32 \times 32 \times 32 \times 64$
		Conv 14	128	$3 \times 3 \times 3$	1	$32 \times 32 \times 32 \times 64$
	2	Upsample 3	-	$2 \times 2 \times 2$	-	$64 \times 64 \times 64 \times 64$
		Conv 15	64	$3 \times 3 \times 3$	1	$64 \times 64 \times 64 \times 32$
		Conv 16	64	$3 \times 3 \times 3$	1	$64 \times 64 \times 64 \times 32$
	1	Upsample 4	-	$2 \times 2 \times 2$	-	$128 \times 128 \times 128 \times 32$
		Conv 17	32	$3 \times 3 \times 3$	1	$128 \times 128 \times 128 \times 16$
		Conv 18	32	$3 \times 3 \times 3$	1	$128 \times 128 \times 128 \times 16$
Output		Conv 19	1	$1 \times 1 \times 1$	1	$128 \times 128 \times 128 \times 1$

Table S4: **CycleGAN generator architecture.** Each residual block (RB) is of the same type as used in VAN-GAN, consisting of of two 3D convolutional layers with a kernel size of $3 \times 3 \times 3$ and stride of $1 \times 1 \times 1$.

	Level	Layer	Filters	Kernel Size	Stride	Output Size
Input						$128 \times 128 \times 128 \times 1$
Downsampling	1	Conv 1	32	$7 \times 7 \times 7$	1	$128 \times 128 \times 128 \times 32$
	2	Conv 2	64	$3 \times 3 \times 3$	2	$64 \times 64 \times 64 \times 64$
	3	Conv 3	128	$3 \times 3 \times 3$	2	$32 \times 32 \times 32 \times 128$
	4	Conv 4	256	$3 \times 3 \times 3$	2	$16 \times 16 \times 16 \times 256$
Residual Blocks	5	RB 1	256	$3 \times 3 \times 3$	1	$16 \times 16 \times 16 \times 256$
	6	RB 2	256	$3 \times 3 \times 3$	1	$16 \times 16 \times 16 \times 256$
	7	RB 3	256	$3 \times 3 \times 3$	1	$16 \times 16 \times 16 \times 256$
	8	RB 4	256	$3 \times 3 \times 3$	1	$16 \times 16 \times 16 \times 256$
	9	RB 5	256	$3 \times 3 \times 3$	1	$16 \times 16 \times 16 \times 256$
	10	RB 6	256	$3 \times 3 \times 3$	1	$16 \times 16 \times 16 \times 256$
Upsampling	11	Upsample 1	128	$2 \times 2 \times 2$	1	$32 \times 32 \times 32 \times 256$
		Conv 5	32	$4 \times 4 \times 4$	1	$32 \times 32 \times 32 \times 128$
	12	Upsample 2	64	$2 \times 2 \times 2$	1	$64 \times 64 \times 64 \times 128$
		Conv 6	32	$4 \times 4 \times 4$	1	$64 \times 64 \times 64 \times 64$
	13	Upsample 3	32	$2 \times 2 \times 2$	1	$128 \times 128 \times 128 \times 64$
		Conv 7	32	$4 \times 4 \times 4$	1	$128 \times 128 \times 128 \times 32$
Output		Conv 8	1	$7 \times 7 \times 7$	1	$128 \times 128 \times 128 \times 1$

Table S5: **Evaluating segmentation performance against 2D maximal intensity projections.** Segmentation masks are calculated for a 3D image using two random forest pixel classifiers (RF) trained separately by two experts (indicated by subscripts *1* and *2*) and our VAN-GAN method. Maximal intensity projections were calculate for each segmentation prediction for a direct comparison against the 2D ground truth masks using F1 Score, intersection over union (IoU), sensitivity and specificity performance metrics. Note, two scores per metric indicates that the metric was computed against the the ground truth generated by user (left) one or (right) two. The highest metric score per ground truth is shown in bold.

Dataset	Method	F1 Score	IoU	Sensitivity	Specificity
**Mouse Ear**	RF_1	**0.658/0.668**	**0.491/0.501**	0.721/0.694	0.905/**0.922**
	RF_2	0.633/0.623	0.463/0.455	**0.785/0.729**	0.860/0.869
	VAN-GAN	0.649/0.630	0.480/0.460	0.688/0.636	**0.912/0.922**
**Mouse Skin**	RF_1	**0.584/0.613**	**0.413/0.443**	0.755/0.711	0.858/0.879
	RF_2	0.562/0.604	0.391/0.434	**0.769/0.739**	0.831/0.855
	VAN-GAN	0.529/0.533	0.360/0.364	0.580/0.532	**0.897/0.911**

Table S6: Mesoscopic photoacoustic image dataset details. Note, voxel size and image dimensions are defined by $X \times Y \times Z$ where the Z-axis is perpendicular to the skin surface. Dataset size indicates the number of image volumes. PA = photoacoustic and PDX = breast cancer patient-derived xenograft.

Dataset	Voxel Size (μm^3)	Image Resolution	Dataset Size	Imaging Modality
**Synthetic**				
Synthetic Vasculature	$20 \times 20 \times 20$	$512 \times 512 \times 140$	449	-
PA Simulations	$20 \times 20 \times 20$	$512 \times 512 \times 140$	449	RSOM
**Experimental**				
Mouse Ear	$20 \times 20 \times 20$	$600 \times 600 \times 140$	32	RSOM
Mouse Skin	$20 \times 20 \times 20$	$600 \times 600 \times 140$	41	RSOM
PDX Tumours	$20 \times 20 \times 20$	$600 \times 600 \times 140$	445	RSOM
MCF7 & MDA-MB-231 Tumours	$20 \times 20 \times 20$	$600 \times 600 \times 140$	204	RSOM
Human Breast	$50 \times 50 \times 50$	$800 \times 800 \times 125$	2	3D-PACT

Table S7: Trained VAN-GAN models with corresponding datasets and workstation specifications used. Workstations are labelled by: 1) a Dell Precision 7920T with a Dual Intel Xeon Gold 5120 CPU with 128GB RAM and two NVIDIA Quadro GV100 32GB GPUs with NVLink; 2) a custom built workstation with a Intel Xeon Gold 5220 CPU with 128GB RAM and four NVIDIA RTX A6000 48GB GPUs with NVLinks. Note, the synthetic vascular dataset is used in all models. PA = photoacoustic and PDX = breast cancer patient-derived xenograft tumours.

VAN-GAN Model	Datasets Used	Workstation	Training Time	Figures
1	PA Simulations	1	54 hours	2<br>4B,C,F,G
2	Mouse Ear<br>Mouse Skin<br>PDX	1	76 hours	3, 4B-G<br>4B-G,<br>5A,B,D
3	Mouse ear<br>Mouse Skin<br>PDX<br>MCF7 & MDA-MB-231	2	36 hours	5F,G,I,J<br>6C-E

References

[1] Lina Hacker, Emma L. Brown, Thierry L. Lefebvre, Paul W. Sweeney, and Sarah E. Bohndiek. Performance evaluation of mesoscopic photoacoustic imaging. *Photoacoustics*, 31:100505, 2023. doi: https://doi.org/10.1016/j.pacs.2023.100505.

[2] Emma L. Brown, Thierry L. Lefebvre, Paul W. Sweeney, Bernadette J. Stolz, Janek Gröhl, Lina Hacker, Ziqiang Huang, Dominique Laurent Couturier, Heather A. Harrington, Helen M. Byrne, and Sarah E. Bohndiek. Quantification of vascular networks in photoacoustic mesoscopy. *Photoacoustics*, 26:100357, 6 2022. ISSN 22135979. doi: 10.1016/j.pacs.2022.100357.

[3] Mithun Kuniyil Ajith Singh and Wiendelt Steenbergen. Photoacoustic-guided focused ultrasound (PAFUSion) for identifying reflection artifacts in photoacoustic imaging. *Photoacoustics*, 3(4):123–131, 12 2015. ISSN 22135979. doi: 10.1016/j.pacs.2015.09.001.

[4] Ho Nhu Y. Nguyen, Altaf Hussain, and Wiendelt Steenbergen. Reflection artifact identification in photoacoustic imaging using multi-wavelength excitation. *Biomedical Optics Express*, 9(10):4613, 10 2018. ISSN 2156-7085. doi: 10.1364/boe.9.004613.

[5] Derek Allman, Austin Reiter, and Muyinatu A.Lediju Bell. Photoacoustic Source Detection and Reflection Artifact Removal Enabled by Deep Learning. *IEEE Transactions on Medical Imaging*, 37 (6):1464–1477, 6 2018. ISSN 1558254X. doi: 10.1109/TMI.2018.2829662.

[6] Ho Nhu Y. Nguyen and Wiendelt Steenbergen. Reducing artifacts in photoacoustic imaging by using multi-wavelength excitation and transducer displacement. *Biomedical Optics Express*, 10(7): 3124, 7 2019. ISSN 2156-7085. doi: 10.1364/boe.10.003124.

[7] James A. Grogan, Anthony J. Connor, Bostjan Markelc, Ruth J. Muschel, Philip K. Maini, Helen M. Byrne, and Joe M. Pitt-Francis. Microvessel Chaste: An Open Library for Spatial Modeling of Vascularized Tissues. *Biophysical Journal*, 112(9):1767–1772, 5 2017. ISSN 15420086. doi: 10.1016/j.bpj.2017.03.036.

[8] Aristid Lindenmayer. Mathematical models for cellular interactions in development I. Filaments with one-sided inputs. *Journal of Theoretical Biology*, 18(3):280–299, 1968. ISSN 10958541. doi: 10.1016/0022-5193(68)90079-9.

[9] Miguel A. Galarreta-Valverde, Maysa M. G. Macedo, Choukri Mekkaoui, and Marcel P. Jackowski. Three-dimensional synthetic blood vessel generation using stochastic L-systems. In *Medical Imaging 2013: Image Processing*, page 86691I, 2013. ISBN 9780819494436. doi: 10.1117/12.2007532.

[10] M. Zamir. Arterial branching within the confines of fractal L-system formalism. *Journal of General Physiology*, 118(3):267–275, 2001. ISSN 00221295. doi: 10.1085/jgp.118.3.267.

[11] Xuemei Liu, Huan Liu, Aimin Hao, and Qinping Zhao. Simulation of blood vessels for surgery simulators. In *2010 International Conference on Machine Vision and Human-Machine Interface, MVHI 2010*, pages 377–380, 2010. ISBN 9780769540092. doi: 10.1109/MVHI.2010.182.
